# Can VEGFC Form Turing Patterns in the Zebrafish Embryo?

**DOI:** 10.1007/s11538-018-00560-2

**Published:** 2019-01-03

**Authors:** Kenneth Y. Wertheim, Tiina Roose

**Affiliations:** 1 Faculty of Engineering and the Environment, University of Southampton, Highfield Campus, Southampton, SO17 1BJ UK; 2 Present Address: University of Nebraska-Lincoln, 1901 Vine St N231, Lincoln, NE 68503 USA

**Keywords:** Reaction–diffusion models, Turing patterns, Lymphangiogenesis, Zebrafish, VEGFC, MMP2, Collagen I

## Abstract

This paper is concerned with a late stage of lymphangiogenesis in the trunk of the zebrafish embryo. At 48 hours post-fertilisation (HPF), a pool of parachordal lymphangioblasts (PLs) lies in the horizontal myoseptum. Between 48 and 168 HPF, the PLs spread from the horizontal myoseptum to form the thoracic duct, dorsal longitudinal lymphatic vessel, and parachordal lymphatic vessel. This paper deals with the potential of vascular endothelial growth factor C (VEGFC) to guide the differentiation of PLs into the mature lymphatic endothelial cells that form the vessels. We built a mathematical model to describe the biochemical interactions between VEGFC, collagen I, and matrix metalloproteinase 2 (MMP2). We also carried out a linear stability analysis of the model and computer simulations of VEGFC patterning. The results suggest that VEGFC can form Turing patterns due to its relations with MMP2 and collagen I, but the zebrafish embryo needs a separate control mechanism to create the right physiological conditions. Furthermore, this control mechanism must ensure that the VEGFC patterns are useful for lymphangiogenesis: stationary, steep gradients, and reasonably fast forming. Generally, the combination of a patterning species, a matrix protein, and a remodelling species is a new patterning mechanism.

## Introduction

**Fig. 1:**
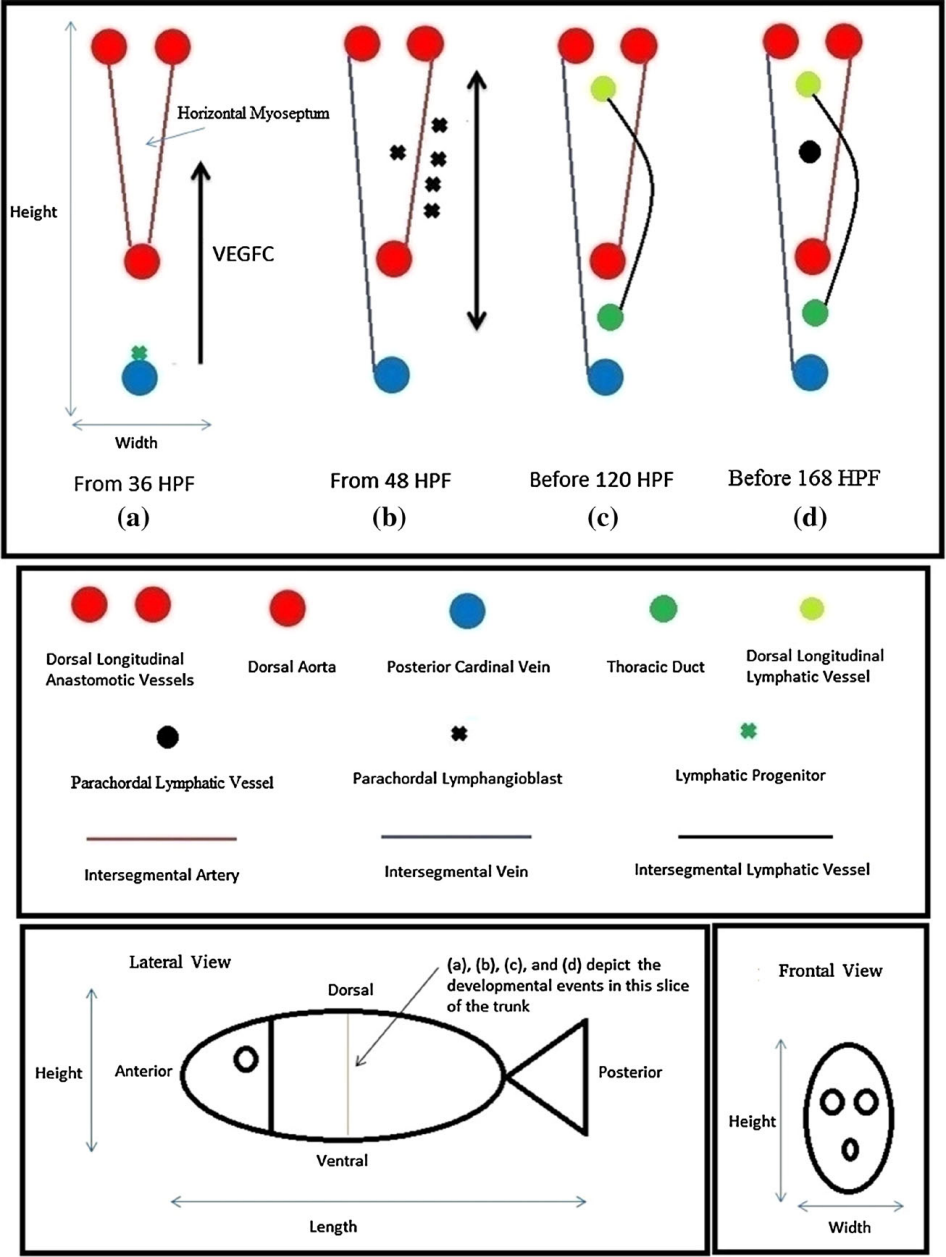
Developmental steps that generate the lymphatic system in the zebrafish embryo’s trunk (Wertheim and Roose 2017); reproduced and modified with permission by the Creative Commons Attribution License. (*a–d*) show a slice of the trunk cut along the ventral–dorsal axis; they depict the developmental events with a frontal view (along the anterior–posterior axis). This slice has a pair of intersegmental arteries (aISVs) and a pair of lymphatic sprouts, one of which fuses with an aISV to form an intersegmental vein (vISV). There are 30 slices like this one in the trunk. (*a*) Under the influence of the vascular endothelial growth factor C (VEGFC), lymphatic progenitor cells exit the posterior cardinal vein. (*b*) They migrate into the horizontal myoseptum to form a pool of parachordal lymphangioblasts (PLs). Eventually, the PLs exit the horizontal myoseptum and migrate along the aISVs. (*c*) When the PLs reach where the thoracic duct and dorsal longitudinal lymphatic vessel lie, they migrate anteriorly and posteriorly to connect with the PLs from the other slices. (*d*) Some lymphatic endothelial cells (resulting from the PLs) leave the intersegmental lymphatic vessel, migrate anteriorly and posteriorly along the horizontal myoseptum, and form the parachordal lymphatic vessel. HPF abbreviates hours post-fertilisation (Color figure online)

**Fig. 2:**
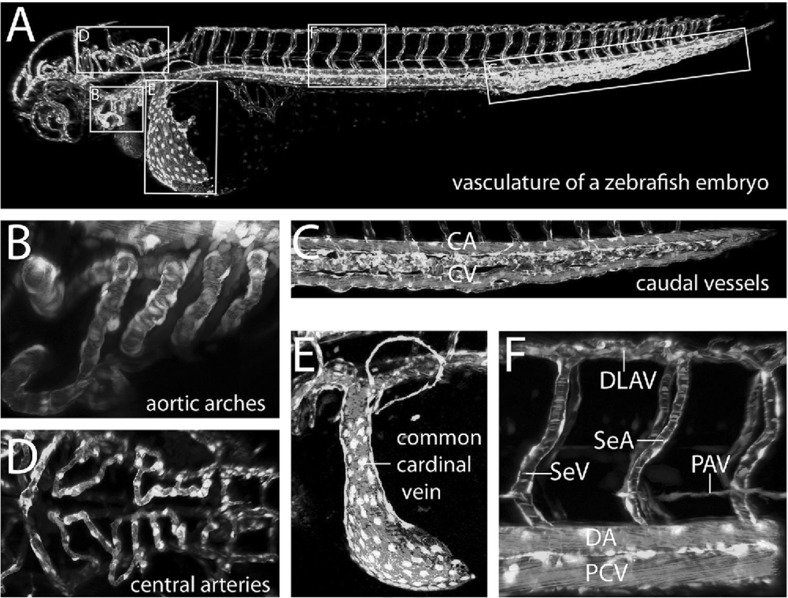
Vascular system in the zebrafish embryo at 60 hours post-fertilisation (Schuermann et al. 2014). Reproduced with permission by a licence provided by Elsevier and Copyright Clearance Center (licence number: 4414440737864, 22/08/2018). The vasculature is shown in green; blood cells, red. **a** Lateral view of the entire vasculature. Magnified versions of the boxed sections are shown in the corresponding subfigures. **b** Lateral view of the aortic arches. **c** Lateral view of the caudal vessels. **d** Bird’s-eye view of the central arteries. **e** Lateral view of the common cardinal vein. **f** Lateral view of a dorsal longitudinal anastomotic vessel (DLAV), an intersegmental vein (vISV or SeV), an intersegmental artery (aISV or SeA), the parachordal vessel (PAV, a pool of parachordal lymphangioblasts in the horizontal myoseptum, not to be confused with the parachordal lymphatic vessel or PLV), the dorsal aorta (DA), and the posterior cardinal vein (PCV) (Color figure online)

We have previously proposed a mathematical model about lymphangiogenesis in the zebrafish embryo’s trunk (Wertheim and Roose 2017); it describes the biochemistry of the process. Specifically, the concentration dynamics of the vascular endothelial growth factor C (VEGFC), matrix metalloproteinase 2 (MMP2), tissue inhibitor of metalloproteinases 2 (TIMP2), and collagen I are described. These dynamics are related to the lymphangiogenic events between 36 and 48 hours post-fertilisation (HPF). These developmental events are illustrated in Fig. 1a, b. In that period, the progenitors of lymphatic endothelial cells (LECs), represented by the green cross in Fig. 1a, exit the posterior cardinal vein (PCV), represented by the blue circle in Fig. 1a, and migrate dorsally to form a pool of parachordal lymphangioblasts (PLs) in the horizontal myoseptum, represented by the black crosses in Fig. 1b. The histological sections in Fig. 2, especially Fig. 2f, help us visualise this sequence of events in the embryo.

This paper is concerned with what happens afterwards. After 48 HPF, the PLs represented by the black crosses in Fig. 1b exit the horizontal myoseptum and migrate both ventrally and dorsally along the intersegmental arteries (aISVs), represented by the red line in Fig. 1b. Before 120 HPF, they form the thoracic duct (TD) and dorsal longitudinal lymphatic vessel (DLLV), represented in Fig. 1c by the dark and light green circles, respectively. As reviewed by Mulligan and Weinstein (2014), some LECs leave the intersegmental lymphatic vessels (ISLVs), represented by the black curve in Fig. 1c. These LECs migrate anteriorly and posteriorly along the horizontal myoseptum to form the parachordal lymphatic vessel (PLV) by 168 HPF; this vessel is represented by the black circle in Fig. 1d.

We speculate that the lymphatic endothelial cells (LECs) in the lymphatic vessels shown in Fig. 1d (dark green, light green, and black circles) have different properties from the parachordal lymphangioblasts (PLs) shown in Fig. 1b (black crosses). First, the LECs can organise into well-defined vessels at their destinations (Coffindaffer-Wilson et al. 2011), as opposed to the transient, nonlumenised string of PLs in the horizontal myoseptum (Hermans et al. 2010). Second, the same pool of PLs leads to at least three different vessels. Functionally, this pool is equivalent to the plexus in the mouse embryo, an initially homogeneous structure which differentiates into lymphatic capillaries, precollectors, and collecting lymphatic vessels (Schulte-Merker et al. 2011).

In summary, the time frame this paper considers is between 48 and 168 HPF. We are interested in what guides the differentiation of the PLs in the horizontal myoseptum into the mature LECs in the TD, DLLV, and PLV.

We hypothesise that VEGFC can form Turing patterns to regulate this process. This classic patterning mechanism was proposed by Turing (1952) and can be defined as follows. When two chemical species with different diffusion rates react with each other, a homogeneous steady state (HSS) may become unstable and give rise to a spatial pattern.

In the next section, we will describe a simplified version of our previous model. After that, we will present a linear stability analysis of the model, focussing on the Turing space; it is the part of the model’s parametric space where Turing’s mechanism is expected to work. Then, we will determine the factors that favour Turing’s mechanism. After analysing computer simulations of the emergence of VEGFC patterns, we will end the paper by relating them to lymphangiogenesis. Due to the large number of abbreviations in this paper, a nomenclature is provided in Table 1.

**Table 1:** Nomenclature

Abbreviations	Meanings
aISV	Intersegmental artery
BDF	Backward differentiation formula
CCBE1	Collagen and calcium-binding EGF domain-containing protein 1
C1	Collagen I
DA	Dorsal aorta
DLAV	Dorsal longitudinal anastomotic vessel
DLLV	Dorsal longitudinal lymphatic vessel
HPF	Hours post-fertilisation
HSS	Homogeneous steady state
ISLV	Intersegmental lymphatic vessel
LEC	Lymphatic endothelial cell
MMP2	Matrix metalloproteinase 2
MT1-MMP	Membrane type I matrix metalloproteinase
ODE	Ordinary differential equation
PCV	Posterior cardinal vein
PL	Parachordal lymphangioblast
PLV	Parachordal lymphatic Vein
proMMP2	Precursor of MMP2
TD	Thoracic duct
TIMP2	Tissue inhibitor of metalloproteinases 2
VEGFC	Vascular endothelial growth factor C
vISV	Intersegmental vein

## Mathematical Model

We will begin this section by describing and justifying the geometry of our model. Our geometry is a line spanning the ‘height’ of the zebrafish trunk. As a first approximation, we decided to consider one dimension only; we could perform a linear stability analysis in one dimension. To keep our model simple, we built it on the ventral–dorsal axis of the zebrafish embryo’s trunk. We made a reasonable approximation because the lymphatic ducts are distributed along that axis and the biochemical gradients in the trunk are along the ventral–dorsal axis (Wertheim and Roose 2017).

Regarding the biochemistry, we drew on our previous work (Wertheim and Roose 2017). In that study, we concluded that VEGFC, MMP2, and collagen I form the axis of the biochemistry underlying lymphangiogenesis. VEGFC is the key regulator of the process; it is the growth factor, and potentially the morphogen and chemotactic factor, for the LECs and their progenitors in the trunk. Collagen I is the major structural component of the trunk; VEGFC binds to it reversibly. MMP2 controls the biophysical properties of the trunk by degrading collagen I. Although TIMP2 binds to MMP2, it only changes the baseline concentration of the latter, not its spatial profile. Therefore, we ignored TIMP2 when we built the model. We also ignored the biochemistry inside the LECs and their progenitors because we were interested in the emergence of VEGFC patterns on the tissue level. In summary, the system is a mass of collagen I bathed in interstitial fluid. Collectively, the mass and fluid constitute the interstitial space; VEGFC and MMP2 are solutes in the interstitial fluid and react with collagen I. VEGFC is the proposed patterning molecule, collagen I controls the transport of VEGFC in the trunk, and MMP2 degrades collagen I.

Regarding the biophysics, we did not model convection because diffusion is the dominant transport phenomenon in the trunk. This is supported by the Péclet number calculated in our previous work: for the diffusivity of VEGFC, the maximum Péclet number is 0.14909 (Wertheim and Roose 2017). VEGFC and MMP2 can diffuse in the interstitial space, but their diffusion rates depend on the abundance of collagen I (Lutter and Makinen 2014). The model describes collagen I as immobile because it is the structural component of this idealised system.

### Model Equations

Inspired by our previous model (Wertheim and Roose 2017), we decided to use a set of reaction–diffusion equations to model the aforementioned biochemical and biophysical events.

In the interstitial space, along the ventral–dorsal axis,

1 $$\begin{aligned} \frac{\partial C_{M2}}{\partial t}= & {} \frac{\partial }{\partial x}\Big [D^{eff}_{M2}\frac{\partial }{\partial x}\Big (\frac{C_{M2}}{\omega }\Big )\Big ] + \frac{P_{M2}C_{VC}C_{C1}}{C_{VC,s}C_{C1,s}}-k^{deg}_{M2}C_{M2}, \end{aligned}$$

2 $$\begin{aligned} \frac{\partial C_{VC}}{\partial t}= & {} \frac{\partial }{\partial x}\Big [D^{eff}_{VC}\frac{\partial }{\partial x}\Big (\frac{C_{VC}}{\omega }\Big )\Big ] + \frac{P_{VC}C_{C1}}{C_{C1,s}}-k^{deg}_{VC}C_{VC}\nonumber \\&-\,k^{on}_{VC,C1}C_{VC}C_{C1}+k^{off}_{VC,C1}C_{VC\cdot C1}, \qquad \end{aligned}$$

3 $$\begin{aligned} \frac{\partial C_{C1}}{\partial t}= & {} P_{C1}-k^{cat}_{M2,C1} C_{M2}-k^{on}_{VC,C1}C_{VC}C_{C1} +k^{off}_{VC,C1}C_{VC\cdot C1},\; \text {and}\qquad \end{aligned}$$

4 $$\begin{aligned} \frac{\partial C_{VC\cdot C1}}{\partial t}= & {} k^{on}_{VC,C1}C_{VC}C_{C1}-k^{off}_{VC,C1}C_{VC\cdot C1}, \end{aligned}$$

where $C_{M2}$ (M) represents the concentration of MMP2; $C_{VC}$ (M) is the concentration of VEGFC; $C_{C1}$ (M) is the concentration of free collagen I; $C_{VC\cdot C1}$ (M) is the concentration of VEGFC-bound collagen I; *t* (s) is time; *x* ($\upmu \hbox {m}$) is the spatial coordinate along the ventral–dorsal axis; $D^{eff}_{i}$ ($\upmu \text {m}^{2}\, \hbox {s}^{-1}$) is the effective diffusivity of species *i*; $\omega$ is the volume fraction of the interstitial space where diffusion occurs; $P_{i}\, (\hbox {M s}^{-1}$) is the production rate of species *i*; $C_{i,s}$ (M) is the concentration scale of species *i*; $k^{deg}_{i} \,(\hbox {s}^{-1}$) is the degradation rate constant of species *i*; $k^{on}_{VC,C1}\, (\hbox {M}^{-1}\, \hbox {s}^{-1}$) is the binding rate constant of VEGFC and collagen I; $k^{off}_{VC,C1}\, (\hbox {s}^{-1}$) is the unbinding rate constant of VEGFC and collagen I; and $k^{cat}_{M2,C1}\, (\hbox {s}^{-1}$) is the MMP2-induced degradation rate constant of collagen I.

In the following subsections, we will explain each term in these equations.

### Diffusion Terms

The diffusion rates of VEGFC and MMP2 are controlled by the abundance of collagen I (Lutter and Makinen 2014). Specifically, they decrease with an increasing concentration of collagen I. This link between transport and kinetics can be found in a paper authored by Ogston et al. (1973):

5 $$\begin{aligned} D^{eff}_{i}=D^{\infty }_{i}\exp \left( \frac{-k_{B}T}{6\pi \mu D^{\infty }_{i}r_{f}}\sqrt{v_{C1}M_{C1}C_{C1}+v_{C1}M_{C1}C_{VC\cdot C1}}\right) , \end{aligned}$$

where $D^{\infty }_{i}\, (\upmu \text {m}^{2} \,\hbox {s}^{-1}$) represents the diffusivity of species *i* in pure interstitial fluid; $k_{B}$ ($1.380648813\times 10^{-23}\hbox { J K }^{-1}$) is the Boltzmann constant; *T* (K) is the temperature in the embryo; $\mu$ (cP) is the dynamic viscosity of interstitial fluid; $r_{f}$ ($\upmu \hbox {m}$) is the radius of a collagen I fibril; and $v_{C1}$ ($\text {cm}^{3} g^{-1}$) is the partial specific volume of dry collagen I.

The volume fraction ($\omega$) where diffusion occurs also depends on the abundance of collagen I (Levick 1987):

6 $$\begin{aligned} \omega =1-v_{C1h}M_{C1}C_{C1}-v_{C1h}M_{C1}C_{VC\cdot C1}, \end{aligned}$$

where $v_{C1h}$ ($\text {cm}^{3}\,\hbox {g}^{-1}$) represents the partial specific volume of hydrated collagen I and $M_{C1}\,(\hbox {kg mol}^{-1}$) is the molar mass of collagen I.

Overall, the diffusive flux of species *i* is in the entire interstitial space, but $\frac{C_{i}}{\omega }$ equals its concentration in the fluid phase only. The effective diffusivity provided by Ogston et al. (1973) converts a concentration gradient in the fluid phase to a flux in the interstitial space.

### Reaction Terms: MMP2

In our idealised system, MMP2 is produced throughout the interstitial space rather than by discrete cells. This simplification is justified because at the developmental stage of interest, the MMP2-producing LECs are scattered around the embryo (Mulligan and Weinstein 2014).

MMP2 is produced inside each LEC by a mechanism that involves the membrane type I matrix metalloproteinase (MT1-MMP), the precursor of MMP2 (proMMP2), and TIMP2 (Karagiannis and Popel 2004). There is evidence of a positive correlation between $C_{VC}$ and $C_{M2}$ (Huang and Sui 2012). There is also evidence that VEGFC increases the level of MT1-MMP (Bauer et al. 2005). Integrating both sources, we speculated that VEGFC upregulates MT1-MMP in order to boost the production of MMP2. Furthermore, there is evidence that collagen I brings MT1-MMP, proMMP2, and TIMP2 closer together (Maquoi et al. 2000), thus boosting MMP2 activation. Integrating these pieces of evidence, we set the production rate of MMP2 to a term proportional to $C_{VC}C_{C1}$.

This production term reflects that VEGFC and collagen I favour MMP2 production through the same mechanism. $P_{M2}$ is the maximum production rate of MMP2; it is achieved when $C_{VC}$ and $C_{C1}$ take their maximum values (their scales). It is possible for collagen I to saturate MT1-MMP when the former is in excess, thereby shutting down MMP2 activation. When we chose this production term, we assumed that the embryo is far from this state.

MMP2 undergoes natural degradation too, hence the degradation term.

### Reaction Terms: VEGFC

In our model, VEGFC is also produced everywhere in the interstitial space. This is justified by the presence of VEGFC-producing aISVs which extend from the dorsal aorta (DA) to the dorsal longitudinal anastomotic vessels (DLAVs) (van Impel and Schulte-Merker 2014).

According to Jeltsch et al. (2014), collagen and calcium-binding EGF domain-containing protein 1 (CCBE1) enhances the secretion and proteolytic cleavage of VEGFC. According to Bos et al. (2011), CCBE1 is likely to act by binding to extracellular matrix components like collagen I. The mechanistic details of this process are unclear. Our production term reflects the little that we do know: collagen I favours VEGFC production. $P_{VC}$ is the maximum production rate of VEGFC. It is achieved when $C_{C1}$ is at its maximum value (its scale).

Similar to MMP2, VEGFC undergoes natural degradation, hence the degradation term.

As we have demonstrated (Wertheim and Roose 2017), the reversible binding between VEGFC and collagen I is an important patterning mechanism for VEGFC.

### Reaction Terms: Collagen I

In our earlier study (Wertheim and Roose 2017), we neglected collagen I production altogether. In that study, we were interested in the transient dynamics of lymphatic development between 36 and 48 HPF. We also assumed that the normal $C_{C1}$ is established prior to that time window. During that window, the migrating LEC progenitors release MMP2 to lower $C_{C1}$ in the embryo.

We built the model presented in this paper in order to study a later developmental stage, specifically the HSS of this stage. As the migration of PLs nears completion, $C_{C1}$ probably rises to its ‘normal’ level. The constant production term models the recovery in $C_{C1}$ and allows a biologically relevant steady state: $C_{C1}>0$ (Swartz and Fleury 2007; Prockop and Kivirikko 1995).

The collagen I degradation term is linear in this model, but we must note that collagen I degradation by MMP2 is enzymatic in nature (Karagiannis and Popel 2004). In our previous study (Wertheim and Roose 2017), we gave this term the form $-\frac{-k^{cat}_{M2,C1}C_{M2}C_{C1}}{K^{M2,C1}_{M}+C_{C1}}$, where $K^{M2,C1}_{M}=8.50\times 10^{-6}$ M. When we decided to linearise this term, we assumed that $C_{C1} \gg K^{M2,C1}_{M}$. According to our previous results (Wertheim and Roose 2017), diffusion is dominant in the zebrafish embryo when $C_{C1}+C_{VC \cdot C1}>1\times 10^{-4}$ M; also, $C_{C1} \gg C_{VC \cdot C1}$. In other words, our assumption is valid as long as diffusion is dominant, i.e. $C_{C1}>1\times 10^{-4}$ M.

We chose not to model collagen I degradation by natural means because its degradation by MMP2 is enzymatic. We assumed that natural degradation is relatively insignificant.

### Reaction Terms: VEGFC-Bound Collagen I

We decided not to model the degradation of VEGFC-bound collagen I, by either MMP2 or natural means.

Although we could find no evidence that MMP2 does not degrade VEGFC-bound collagen I, we decided not to model the degradation of the latter. First, due to steric effects, MMP2 is likely to target free collagen I fibrils over those obstructed by VEGFC. Second, we wanted to keep the mathematics as simple as possible. Third, our model does describe the MMP2-induced degradation dynamics of VEGFC-bound collagen I indirectly. According to the model, when the concentration of collagen I decreases, the production term for VEGFC-bound collagen I will decrease, thus increasing the net dissociation rate of VEGFC-bound collagen I. Once released, collagen I and VEGFC can degrade through MMP2 and natural means, respectively.

With respect to natural degradation, the reasoning is along the same lines. First, when VEGFC is sequestered by collagen I, it is less exposed to the molecular species involved in its natural degradation. Second, when the concentration of VEGFC decreases due to natural degradation, the production term for VEGFC-bound collagen I will go down, and the net dissociation rate of VEGFC-bound collagen I will go up. Once released, VEGFC can degrade naturally.

### Boundary and Initial Conditions

We denote the embryo’s height by *L* (µm). The boundary conditions where $x=0$ and $x=L$ are given by the following no-flux boundary conditions:

7 $$\begin{aligned}&\displaystyle \frac{\partial }{\partial x}\left( \frac{C_{M2}}{\omega }\right) =0 \; \text {and} \end{aligned}$$

8 $$\begin{aligned}&\displaystyle \frac{\partial }{\partial x}\left( \frac{C_{VC}}{\omega }\right) =0. \end{aligned}$$

We wanted to study the behaviour of the HSS in the presence of thermal noises. Therefore, we set each initial concentration to the HSS concentration plus a stochastic term.

### Parametrisation and Nondimensionalisation

Most of the parametric values can be found in our previous study (Wertheim and Roose 2017). However, three parameters require special attention.

For $P_{M2}$, we chose the rate of proMMP2 production by lymphatic progenitors ($2.64\times 10^{-8}\hbox { M s}^{-1}$) from our previous study (Wertheim and Roose 2017; Vempati et al. 2010). The current model does not consider the intracellular activation of proMMP2, so in choosing to adopt the proMMP2 value, we assumed that the rate of proMMP2 production is equal to the rate of MMP2 activation. Clearly, in doing so, we ignored many intermediate steps. However, we chose a reasonable starting point at which a sensitivity analysis was carried out (discussed in Sect. 4.5); it is the upper limit of $P_{M2}$.

For $P_{VC}$, we chose the rate of VEGFC production on the dorsal aorta surface ($1.65\times 10^{-17}\hbox { mol dm}^{-2} \, \hbox {s}^{-1}$) from our previous study (Wertheim and Roose 2017; Hashambhoy et al. 2011). In the current model, VEGFC is produced throughout the embryo and not just on the dorsal aorta surface. Assuming a cell diameter of 10 $\upmu \hbox {m}$, we converted it to $9.90\times 10^{-13}\hbox { M s}^{-1}$. Once again, this is a crude estimate. As we will discuss in Sect. 4.5, we performed a sensitivity analysis on this parameter.

We calculated the value of $P_{C1}$ by nondimensionalising the model because this parameter depends on the scale of $C_{C1}$.

The length scale is the height of the trunk because our geometry is a cutline along the ventral–dorsal axis, so $L=434$ µm (McGee et al. 2012). We picked a timescale ($\tau$) of 10000 s, the timescale of natural degradation of VEGFC and MMP2. As explained at the beginning of this section, our concern was the diffusion-dominant regime and we had established that diffusion dominates convection when $C_{C1}=5.29\times 10^{-4}$ M (Wertheim and Roose 2017). We chose this value for $C_{C1,s}$.

We determined the remaining concentration scales ($C_{i,s}$’s) and $P_{C1}$ by finding the HSS where $C_{C1}=C_{C1,s}$. Neglecting the spatial and temporal variations modelled by Eqs. (1)–(4), we obtained the HSS in terms of a set of algebraic equations. We solved them for the concentration scales and $P_{C1}$. The results are as follows:

9 $$\begin{aligned}&\displaystyle C_{M2,s}= \frac{P_{M2}}{k^{deg}_{M2}}, \end{aligned}$$

10 $$\begin{aligned}&\displaystyle C_{VC,s}= \frac{P_{VC}}{k^{deg}_{VC}}, \end{aligned}$$

11 $$\begin{aligned}&\displaystyle P_{C1}=k^{cat}_{M2,C1}C_{M2,s},\; \text {and}\end{aligned}$$

12 $$\begin{aligned}&\displaystyle C_{VC\cdot C1,s}=\frac{k^{on}_{VC,C1}C_{VC,s}C_{C1,s}}{k^{off}_{VC,C1}}. \end{aligned}$$

Numerically, $C_{VC,s}=9.90\times 10^{-9}$ M, $C_{M2,s}=2.64\times 10^{-4}$ M, $P_{C1}=1.19\times 10^{-6}\hbox { M s}^{-1}$, and $C_{VC \cdot C1,s}=5.24\times 10^{-5}$ M. The characteristic scales of the model are summarised in Table 2. We set $P_{C1}$ at $6\times 10^{-7}\hbox { M s}^{-1}$. As a result, $C_{C1}$ stays within its scale at the HSS. Because the production rates of VEGFC and MMP2 scale linearly with $C_{C1}$, the other concentrations ($C_{VC}$, $C_{M2}$, and $C_{VC\cdot C1}$) should stay within their scales at the HSS too.

**Table 2:** Characteristic scales of the model

Scale	Description	Value
$C_{C1,s}$	Concentration scale for C1	$5.29\times 10^{-4}$ M
$C_{M2,s}$	Concentration scale for M2	$2.64\times 10^{-4}$ M
$C_{VC,s}$	Concentration scale for VC	$9.90\times 10^{-9}$ M
$C_{VC \cdot C1,s}$	Concentration scale for VC$\cdot$C1	$5.24\times 10^{-5}$ M
*L*	Length scale	434 $\upmu \hbox {m}$
$\tau$	Timescale	10,000 s

M2 abbreviates MMP2; VC, VEGFC; C1, collagen I

We nondimensionalised equations (1)–(4) using the established length, time, and concentration scales, i.e. $$L=434$$$$\upmu \hbox {m}$$, $$\tau =10000$$ s, $$C_{C1,s}=5.29\times 10^{-4}$$ M, $$C_{VC,s}=9.90\times 10^{-9}$$ M, $$C_{M2,s}=2.64\times 10^{-4}$$ M, and $$C_{VC \cdot C1,s}=5.24\times 10^{-5}$$ M. The nondimensionalised model is as follows:

13 $$\begin{aligned} \frac{\partial {\tilde{C}}_{M2}}{\partial {\tilde{t}}}= & {} \frac{\partial }{\partial \tilde{x}}\left[ a_{1,M2}\exp \left( -a_{2,M2}\sqrt{a_{3}{\tilde{C}}_{C1} +\frac{a_{3}b_{4}}{b_{5}}{\tilde{C}}_{VC \cdot C1}}\right) \right. \nonumber \\&\left. \frac{\partial }{\partial \tilde{x}}\left( \frac{{\tilde{C}}_{M2}}{1-a_{4}{\tilde{C}}_{C1} -\frac{a_{4}b_{4}}{b_{5}}{\tilde{C}}_{VC \cdot C1}}\right) \right] +{\tilde{C}}_{VC}{\tilde{C}}_{C1}-{\tilde{C}}_{M2}, \end{aligned}$$

14 $$\begin{aligned} \frac{\partial {\tilde{C}}_{VC}}{\partial {\tilde{t}}}= & {} \frac{\partial }{\partial \tilde{x}}\left[ a_{1,VC}\exp \left( -a_{2,VC}\sqrt{a_{3} {\tilde{C}}_{C1}+\frac{a_{3}b_{4}}{b_{5}}{\tilde{C}}_{VC \cdot C1}}\right) \right. \nonumber \\&\left. \frac{\partial }{\partial \tilde{x}}\left( \frac{{\tilde{C}}_{VC}}{1-a_{4}{\tilde{C}}_{C1} -\frac{a_{4}b_{4}}{b_{5}}{\tilde{C}}_{VC \cdot C1}}\right) \right] \nonumber \\&+\,{\tilde{C}}_{C1}-{\tilde{C}}_{VC}-b_{1}({\tilde{C}}_{VC}{\tilde{C}}_{C1} -{\tilde{C}}_{VC \cdot C1}), \end{aligned}$$

15 $$\begin{aligned}&\displaystyle \frac{\partial {\tilde{C}}_{C1}}{\partial {\tilde{t}}}=b_{2}-b_{3}{\tilde{C}}_{M2}-b_{4}({\tilde{C}}_{VC} {\tilde{C}}_{C1}-{\tilde{C}}_{VC \cdot C1}),\; \text {and} \end{aligned}$$

16 $$\begin{aligned}&\displaystyle \frac{\partial {\tilde{C}}_{VC \cdot C1}}{\partial {\tilde{t}}}=b_{5}({\tilde{C}}_{VC}{\tilde{C}}_{C1}-{\tilde{C}}_{VC \cdot C1}). \end{aligned}$$

The boundary conditions where $\tilde{x}=0$ and $\tilde{x}=1$ are given by the equations,

17 $$\begin{aligned}&\displaystyle \frac{\partial }{\partial \tilde{x}}\left( \frac{{\tilde{C}}_{M2}}{1-a_{4}{\tilde{C}}_{C1} -\frac{a_{4}b_{4}}{b_{5}}{\tilde{C}}_{VC \cdot C1}}\right) =0 \; \text {and} \end{aligned}$$

18 $$\begin{aligned}&\displaystyle \frac{\partial }{\partial \tilde{x}}\left( \frac{{\tilde{C}}_{VC}}{1-a_{4}{\tilde{C}}_{C1} -\frac{a_{4}b_{4}}{b_{5}}{\tilde{C}}_{VC \cdot C1}}\right) =0. \end{aligned}$$

There are fewer dimensionless kinetic parameters than dimensional ones. It is because we have chosen the scales such that $k^{deg}_{M2}\tau =k^{deg}_{VC}\tau =1$. The dimensionless parameters ($a_{i}$’s and $b_{i}$’s) are summarised in Table 3, while the dimensional parameters that constitute them are summarised in Table 4. Mathematically, $a_{1,M2}=\frac{D^{\infty }_{M2}\tau }{L^{2}}$, $a_{1,VC}=\frac{D^{\infty }_{VC}\tau }{L^{2}}$, $a_{2,M2}=\frac{k_{B}T}{6\pi \mu D^{\infty }_{M2} r_{f}}$, $a_{2,VC}=\frac{k_{B}T}{6\pi \mu D^{\infty }_{VC} r_{f}}$, $a_{3}=v_{C1}M_{C1}C_{C1,s}$, $a_{4}=v_{C1h}M_{C1}C_{C1,s}$, $b_{1}=k^{on}_{VC,C1}\tau C_{C1,s}$, $b_{2}=\frac{P_{C1}\tau }{C_{C1,s}}$, $b_{3}=\frac{k^{cat}_{M2,C1}\tau P_{M2}}{k^{deg}_{M2}C_{C1,s}}$, $b_{4}=\frac{k^{on}_{VC,C1}\tau P_{VC}}{k^{deg}_{VC}}$, $b_{5}=k^{off}_{VC,C1}\tau$. In addition, a variable with a tilde is nondimensionalised. Therefore, $\tilde{x}$ is $\frac{x}{L}$ or the nondimensionalised length in the *x*-direction; ${\tilde{t}}$ is $\frac{t}{\tau }$ or the nondimensionalised *t*; ${\tilde{C}}_{C1}$ is $\frac{C_{C1}}{C_{C1,s}}$ or the nondimensionalised $C_{C1}$; ${\tilde{C}}_{M2}$ is $\frac{C_{M2}}{C_{M2,s}}$ or the nondimensionalised $C_{M2}$; ${\tilde{C}}_{VC}$ is $\frac{C_{VC}}{C_{VC,s}}$ or the nondimensionalised $C_{VC}$; ${\tilde{C}}_{VC \cdot C1}$ is $\frac{C_{VC \cdot C1}}{C_{VC \cdot C1,s}}$ or the nondimensionalised $C_{VC \cdot C1}$.

**Table 3:** Dimensionless parameters in the nondimensionalised model

Parameter	Form	Value
$b_{1}$	$k^{on}_{VC,C1}\tau C_{C1,s}$	$1.90\times 10^{5}$
$b_{2}$	$\frac{P_{C1}\tau }{C_{C1,s}}$	$1.13\times 10^{1}$
$b_{3}$	$\frac{k^{cat}_{M2,C1}\tau P_{M2}}{k^{deg}_{M2}C_{C1,s}}$	$2.25\times 10^{1}$
$b_{4}$	$\frac{k^{on}_{VC,C1}\tau P_{VC}}{k^{deg}_{VC}}$	3.56
$b_{5}$	$k^{off}_{VC,C1}\tau$	$3.60\times 10^{1}$
$a_{1,M2}$	$\frac{D^{\infty }_{M2}\tau }{L^{2}}$	4.51
$a_{1,VC}$	$\frac{D^{\infty }_{VC}\tau }{L^{2}}$	2.66
$a_{2,M2}$	$\frac{k_{B}T}{6\pi \mu D^{\infty }_{M2} r_{f}}$	1.07
$a_{2,VC}$	$\frac{k_{B}T}{6\pi \mu D^{\infty }_{VC} r_{f}}$	1.81
$a_{3}$	$v_{C1}M_{C1}C_{C1,s}$	$1.19\times 10^{-1}$
$a_{4}$	$v_{C1h}M_{C1}C_{C1,s}$	$3.00\times 10^{-1}$

M2 abbreviates MMP2; VC, VEGFC; C1, collagen I

**Table 4:** Dimensional parameters that constitute the dimensionless parameters in the nondimensionalised model

Parameter	Definition	Value
$P_{C1}$	Production rate of C1	$6.00\times 10^{-7}\hbox { M s}^{-1}$
$P_{M2}$	Production rate of M2	$2.64\times 10^{-8}\hbox { M s}^{-1}$
$P_{VC}$	Production rate of VC	$9.90\times 10^{-13}\hbox { M s}^{-1}$
$k^{on}_{VC,C1}$	Binding rate constant of VC and C1	$3.60\times 10^{4} \, \hbox {M}^{-1}\, \hbox {s}^{-1}$
$k^{off}_{VC,C1}$	Unbinding rate constant of VC and C1	$3.60\times 10^{-3} \, \hbox {s}^{-1}$
$k^{cat}_{M2,C1}$	Turnover number in the degradation of C1 by M2	$4.50\times 10^{-3} \, \hbox {s}^{-1}$
$k^{deg}_{M2}$	Degradation rate constant of M2	$1.00\times 10^{-4} \, \hbox {s}^{-1}$
$k^{deg}_{VC}$	Degradation rate constant of VC	$1.00\times 10^{-4} \, \hbox {s}^{-1}$
$D^{\infty }_{M2}$	Diffusivity of M2	$8.50\times 10^{-7}\, \hbox {cm}^{2} \, \hbox {s}^{-1}$
$D^{\infty }_{VC}$	Diffusivity of VC	$5.01\times 10^{-7}\, \hbox {cm}^{2} \, \hbox {s}^{-1}$
$M_{C1}$	Molecular weight of a collagen I fibril	$300\hbox { kg mol}^{-1}$
$r_{f}$	Radius of a C1 fibril	2 nm
$v_{C1}$	Specific volume of dry C1	0.75 $\hbox {cm}^{3} \, \hbox {g}^{-1}$
$v_{C1h}$	Specific volume of hydrated C1	1.89 $\hbox {cm}^{3} \, \hbox {g}^{-1}$
$k_{B}$	Boltzmann constant	$1.38\times 10^{-23}\hbox { J K}^{-1}$
*T*	Temperature	298 K
$\mu$	Dynamic viscosity of interstitial fluid	1.20 cP

M2 abbreviates MMP2; VC, VEGFC; C1, collagen I

## Linear Stability Analysis

The model presented in this paper differs from the classic instance of Turing’s mechanism (Gierer and Meinhardt 1972) in three ways. First, VEGFC and MMP2 do not form a self-activator-self-inhibitor pair as defined by Gierer and Meinhardt (1972). While VEGFC stimulates MMP2 production, it does not stimulate its own production. MMP2 does not inhibit VEGFC production either. However, MMP2 degrades collagen I, thereby inhibiting the production of both species indirectly. Second, VEGFC binds to an immobile substrate (collagen I) reversibly. Third, the diffusion rates of both VEGFC and MMP2 depend on the concentration of collagen I. This section is concerned with the criteria for Turing’s mechanism to work in this atypical system.

### Homogeneous Steady State

A Turing pattern emerges from a homogeneous steady state (HSS) by definition. Of course, a genuine steady state is impossible due to thermal noises. Nonetheless, we argue that the biochemical profile of the zebrafish trunk is sufficiently close to a HSS in our time frame of interest. First, at this late developmental stage, the cells producing MMP2 are scattered around the embryo. Second, the VEGFC-producing aISVs span the embryo along its ventral–dorsal axis. Third, diffusion dominates convection in this model.

After building and nondimensionalising the model, our first step was to determine its HSS. We set the derivatives in Eqs. (13)–(16) to zero, thereby obtaining a set of algebraic equations. Denoting the steady-state concentration of species *i* by ${\tilde{C}}_{i,ss}$, we can write down the algebraic equations,

19 $$\begin{aligned} 0= & {} {\tilde{C}}_{VC,ss}{\tilde{C}}_{C1,ss}-{\tilde{C}}_{M2,ss}, \end{aligned}$$

20 $$\begin{aligned} 0= & {} {\tilde{C}}_{C1,ss}-{\tilde{C}}_{VC,ss}-b_{1}({\tilde{C}}_{VC,ss} {\tilde{C}}_{C1,ss}-{\tilde{C}}_{VC \cdot C1,ss}), \end{aligned}$$

21 $$\begin{aligned} 0= & {} b_{2}-b_{3}{\tilde{C}}_{M2,ss}-b_{4}({\tilde{C}}_{VC,ss}{\tilde{C}}_{C1,ss} -{\tilde{C}}_{VC \cdot C1,ss}),\; \text {and} \end{aligned}$$

22 $$\begin{aligned} 0= & {} {\tilde{C}}_{VC,ss}{\tilde{C}}_{C1,ss}-{\tilde{C}}_{VC \cdot C1,ss}. \end{aligned}$$

Their solution gives the following: ${\tilde{C}}_{M2,ss}=\frac{b_{2}}{b_{3}}$, ${\tilde{C}}_{VC,ss}=\sqrt{\frac{b_{2}}{b_{3}}}$, ${\tilde{C}}_{C1,ss}=\sqrt{\frac{b_{2}}{b_{3}}}$, and ${\tilde{C}}_{VC \cdot C1,ss}=\frac{b_{2}}{b_{3}}$. Since $b_{2}$ and $b_{3}$ are positive, the steady-state concentrations are positive and physical. However, the model must satisfy two conditions. First, ${\tilde{C}}_{C1,ss}>1.89\times 10^{-1}$ M, so the HSS stays in the diffusion-dominant regime. Second, $b_{2}$ must be smaller than $b_{3}$ to ensure that the concentrations are between 0 and 1.

### Homogeneous Perturbation

Turing’s mechanism relies on diffusion-driven instability (Turing 1952). A HSS must be stable in response to small perturbations in time only. When spatial perturbations are present, diffusion amplifies certain components of the perturbations to create a pattern.

To check the stability of the HSS without diffusion, we neglected the diffusion terms in Eqs. (13)–(16). Then, we performed a linear stability analysis on the remaining ordinary differential equations (ODEs). In other words, we expanded the right-hand side of each ODE as a Taylor series at the HSS and neglected the nonlinear, higher-order terms. We will denote the perturbation to ${\tilde{C}}_{i,ss}$ by $\Delta {\tilde{C}}_{i}$ such that ${\tilde{C}}_{i}= {\tilde{C}}_{i,ss}+\Delta {\tilde{C}}_{i}$. After the said expansion and elimination, we obtained the following system of ODEs:

23 $$\begin{aligned} \frac{\partial }{\partial {\tilde{t}}} \begin{pmatrix} \Delta {\tilde{C}}_{M2}\\ \Delta {\tilde{C}}_{VC}\\ \Delta {\tilde{C}}_{C1}\\ \Delta {\tilde{C}}_{VC \cdot C1} \end{pmatrix}= \begin{pmatrix} -1 &{} {\tilde{C}}_{C1,ss} &{} {\tilde{C}}_{VC,ss} &{} 0\\ 0 &{} -1-b_{1}{\tilde{C}}_{C1,ss} &{} 1-b_{1}{\tilde{C}}_{VC,ss} &{} b_{1}\\ -b_{3} &{} -b_{4}{\tilde{C}}_{C1,ss} &{} -b_{4}{\tilde{C}}_{VC,ss} &{} b_{4}\\ 0 &{} b_{5}{\tilde{C}}_{C1,ss} &{} b_{5}{\tilde{C}}_{VC,ss} &{} -b_{5}\\ \end{pmatrix} \begin{pmatrix} \Delta {\tilde{C}}_{M2}\\ \Delta {\tilde{C}}_{VC}\\ \Delta {\tilde{C}}_{C1}\\ \Delta {\tilde{C}}_{VC \cdot C1} \end{pmatrix}. \end{aligned}$$

We will name the square matrix in Eq. (23) *A* and its eigenvalues $\sigma$’s. The eigenvalues are the solutions of this equation: $$\begin{vmatrix} A-\sigma I\end{vmatrix}=0$$ (*I* is the identity matrix). This quartic equation has four solutions. If all four eigenvalues have negative real parts, $\Delta {\tilde{C}}_{i}$’s approach zero as ${\tilde{t}}$ approaches infinity, meaning the HSS is stable to noises in the absence of diffusion.

Therefore, the third constraint the model must satisfy is this: the eigenvalues of the square matrix *A* must have negative real parts.

**Fig. 3:**
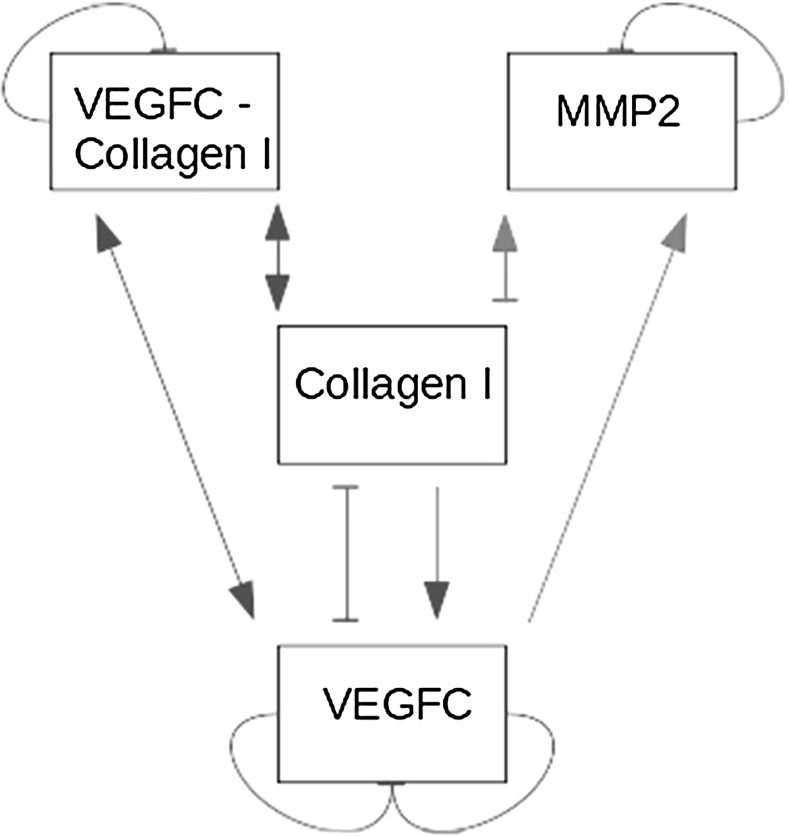
Linearised interaction map representing the relations between VEGFC, MMP2, collagen I, and VEGFC-bound collagen I. A pointy edge means an activating relation; for example, VEGFC activates MMP2. A blunt edge means an inhibitory relation; for example, MMP2 inhibits collagen I. The blue interactions are all related to the reversible binding reaction between VEGFC and collagen I. The red ones are related to the production of MMP2 to degrade collagen I. The green ones are natural degradation reactions. VEGFC stands for vascular endothelial growth factor C; MMP2, matrix metalloproteinase 2 (Color figure online)

Before we continue, it is worth visualising the linearised interactions represented by *A*. They are plotted in Fig. 3. Each edge represents a term in *A*, wherein two matrix elements have two terms apiece. First, according to Fig. 3, VEGFC inhibits itself in two ways; it degrades naturally and complexes with collagen I. Second, collagen I stimulates VEGFC production and complexes with it, so there are two terms in the matrix element about the influence of collagen I on VEGFC.

There are two control loops in Fig. 3. In the model, there is a constant supply of collagen I, which stimulates VEGFC production; once produced, VEGFC and collagen I bind to prevent degradation, while more collagen I and VEGFC are produced; meanwhile, VEGFC-bound collagen I dissociates to increase the supply of both. This positive feedback loop raises the production rate of VEGFC over time. It is balanced by a negative feedback loop. VEGFC stimulates the production of MMP2, which degrades collagen I to suppress the positive feedback loop.

### Heterogeneous Perturbation

Next, we studied the HSS’s stability in the presence of random noises and diffusion.

First, we linearised the diffusion terms in Eqs. (13) and (14). They have the form $\frac{\partial }{\partial \tilde{x}}\Big [a_{1,i}\exp \Big (-a_{2,i}\sqrt{a_{3}{\tilde{C}}_{C1}+\frac{a_{3}b_{4}}{b_{5}}{\tilde{C}}_{VC \cdot C1}}\Big )\frac{\partial }{\partial \tilde{x}}\Big (\frac{{\tilde{C}}_{i}}{1-a_{4}{\tilde{C}}_{C1}-\frac{a_{4}b_{4}}{b_{5}}{\tilde{C}}_{VC \cdot C1}}\Big )\Big ]$. As in the last subsection, we will denote the perturbation to ${\tilde{C}}_{i,ss}$ by $\Delta {\tilde{C}}_{i}$ such that ${\tilde{C}}_{i}= {\tilde{C}}_{i,ss}+\Delta {\tilde{C}}_{i}$.

We expanded $a_{1,i}\exp \Big (-a_{2,i}\sqrt{a_{3}{\tilde{C}}_{C1}+\frac{a_{3}b_{4}}{b_{5}}{\tilde{C}}_{VC \cdot C1}}\Big )$ at the HSS to obtain a Taylor series, truncated the series, and obtained this approximation:

24 $$\begin{aligned}&a_{1,i}\exp \left( -a_{2,i}\sqrt{a_{3}{\tilde{C}}_{C1}+\frac{a_{3}b_{4}}{b_{5}}{\tilde{C}}_{VC \cdot C1}}\right) \approx a_{1,i}\exp \left( -a_{2,i}\sqrt{a_{3}{\tilde{C}}_{C1,ss}+\frac{a_{3}b_{4}}{b_{5}}{\tilde{C}}_{VC \cdot C1,ss}}\right) \nonumber \\&\quad +\,a_{1,i}\exp \left( -a_{2,i}\sqrt{a_{3}{\tilde{C}}_{C1,ss}+\frac{a_{3}b_{4}}{b_{5}}{\tilde{C}}_{VC \cdot C1,ss}}\right) \left[ \frac{-a_{2,i}a_{3}}{2\sqrt{a_{3}{\tilde{C}}_{C1,ss}+\frac{a_{3}b_{4}}{b_{5}}{\tilde{C}}_{VC \cdot C1,ss}}}\right] \Delta {\tilde{C}}_{C1}\nonumber \\&\quad +\,a_{1,i}\exp \left( -a_{2,i}\sqrt{a_{3}{\tilde{C}}_{C1,ss}+\frac{a_{3}b_{4}}{b_{5}}{\tilde{C}}_{VC \cdot C1,ss}}\right) \nonumber \\&\quad \left[ \frac{-a_{2,i}a_{3}b_{4}}{2b_{5}\sqrt{a_{3}{\tilde{C}}_{C1,ss}+\frac{a_{3}b_{4}}{b_{5}}{\tilde{C}}_{VC \cdot C1,ss}}}\right] \Delta {\tilde{C}}_{VC \cdot C1}. \end{aligned}$$

Doing the same for $\frac{{\tilde{C}}_{i}}{1-a_{4}{\tilde{C}}_{C1}-\frac{a_{4}b_{4}}{b_{5}}{\tilde{C}}_{VC \cdot C1}}$, we arrived at the following:

25 $$\begin{aligned}&\frac{{\tilde{C}}_{i}}{1-a_{4}{\tilde{C}}_{C1}-\frac{a_{4}b_{4}}{b_{5}}{\tilde{C}}_{VC \cdot C1}} \approx \frac{{\tilde{C}}_{i,ss}}{1-a_{4}{\tilde{C}}_{C1,ss}-\frac{a_{4}b_{4}}{b_{5}}{\tilde{C}}_{VC \cdot C1,ss}}\nonumber \\&\quad +\,\frac{\Delta {\tilde{C}}_{i}}{1-a_{4}{\tilde{C}}_{C1,ss}-\frac{a_{4}b_{4}}{b_{5}}{\tilde{C}}_{VC \cdot C1,ss}}+\frac{{\tilde{C}}_{i,ss}a_{4}\Delta {\tilde{C}}_{C1}}{(1-a_{4}{\tilde{C}}_{C1,ss}-\frac{a_{4}b_{4}}{b_{5}}{\tilde{C}}_{VC \cdot C1,ss})^{2}}\nonumber \\&\quad +\,\frac{{\tilde{C}}_{i,ss}a_{4}b_{4}\Delta {\tilde{C}}_{VC \cdot C1}}{b_{5}(1-a_{4}{\tilde{C}}_{C1,ss}-\frac{a_{4}b_{4}}{b_{5}}{\tilde{C}}_{VC \cdot C1,ss})^{2}}. \end{aligned}$$

Substituting these two series into the general diffusion term, $\frac{\partial }{\partial \tilde{x}}\Big [a_{1,i}\exp \Big (-a_{2,i}\sqrt{a_{3}{\tilde{C}}_{C1}+\frac{a_{3}b_{4}}{b_{5}}{\tilde{C}}_{VC \cdot C1}}\Big )\frac{\partial }{\partial \tilde{x}}\Big (\frac{{\tilde{C}}_{i}}{1-a_{4}{\tilde{C}}_{C1}-\frac{a_{4}b_{4}}{b_{5}}{\tilde{C}}_{VC \cdot C1}}\Big )\Big ]$, ignoring the nonlinear terms, we obtained the following results for the diffusion term:

26 $$\begin{aligned}&\frac{\partial }{\partial \tilde{x}}\left[ a_{1,i}\exp \left( -a_{2,i}\sqrt{a_{3}{\tilde{C}}_{C1}+\frac{a_{3}b_{4}}{b_{5}}{\tilde{C}}_{VC \cdot C1}}\right) \frac{\partial }{\partial \tilde{x}}\left( \frac{{\tilde{C}}_{i}}{1-a_{4}{\tilde{C}}_{C1}-\frac{a_{4}b_{4}}{b_{5}}{\tilde{C}}_{VC \cdot C1}}\right) \right] \nonumber \\&\quad \approx \frac{a_{1,i}\exp \left( -a_{2,i}\sqrt{a_{3}{\tilde{C}}_{C1,ss}+\frac{a_{3}b_{4}}{b_{5}}{\tilde{C}}_{VC \cdot C1,ss}}\right) }{1-a_{4}{\tilde{C}}_{C1,ss}-\frac{a_{4}b_{4}}{b_{5}}{\tilde{C}}_{VC \cdot C1,ss}}\frac{\partial ^{2}\Delta {\tilde{C}}_{i}}{\partial \tilde{x}^{2}}\nonumber \\&\qquad +\,\frac{a_{1,i}\exp \left( -a_{2,i}\sqrt{a_{3}{\tilde{C}}_{C1,ss}+\frac{a_{3}b_{4}}{b_{5}}{\tilde{C}}_{VC \cdot C1,ss}}\right) a_{4}{\tilde{C}}_{i,ss}}{(1-a_{4}{\tilde{C}}_{C1,ss}-\frac{a_{4}b_{4}}{b_{5}}{\tilde{C}}_{VC \cdot C1,ss})^{2}}\frac{\partial ^{2}\Delta {\tilde{C}}_{C1}}{\partial \tilde{x}^{2}}\nonumber \\&\qquad +\,\frac{a_{1,i}\exp \left( -a_{2,i}\sqrt{a_{3}{\tilde{C}}_{C1,ss}+\frac{a_{3}b_{4}}{b_{5}}{\tilde{C}}_{VC \cdot C1,ss}}\right) a_{4}b_{4}{\tilde{C}}_{i,ss}}{(1-a_{4}{\tilde{C}}_{C1,ss}-\frac{a_{4}b_{4}}{b_{5}}{\tilde{C}}_{VC \cdot C1,ss})^{2}b_{5}}\frac{\partial ^{2}\Delta {\tilde{C}}_{VC \cdot C1}}{\partial \tilde{x}^{2}}. \end{aligned}$$

Equation (26) has a simpler form in terms of the following definitions:

27 $$\begin{aligned} d_{1,i}= & {} \frac{a_{1,i}\exp \Big (-a_{2,i}\sqrt{a_{3}{\tilde{C}}_{C1,ss} +\frac{a_{3}b_{4}}{b_{5}}{\tilde{C}}_{VC \cdot C1,ss}}\Big )}{1-a_{4}{\tilde{C}}_{C1,ss} -\frac{a_{4}b_{4}}{b_{5}}{\tilde{C}}_{VC \cdot C1,ss}}, \end{aligned}$$

28 $$\begin{aligned} d_{2,i}= & {} \frac{a_{1,i}\exp \Big (-a_{2,i}\sqrt{a_{3}{\tilde{C}}_{C1,ss} +\frac{a_{3}b_{4}}{b_{5}}{\tilde{C}}_{VC \cdot C1,ss}}\Big )a_{4}{\tilde{C}}_{i,ss}}{(1-a_{4}{\tilde{C}}_{C1,ss} -\frac{a_{4}b_{4}}{b_{5}}{\tilde{C}}_{VC \cdot C1,ss})^{2}},\; \text {and} \end{aligned}$$

29 $$\begin{aligned} d_{3,i}= & {} \frac{a_{1,i}\exp \Big (-a_{2,i}\sqrt{a_{3}{\tilde{C}}_{C1,ss} +\frac{a_{3}b_{4}}{b_{5}}{\tilde{C}}_{VC \cdot C1,ss}}\Big )a_{4}b_{4}{\tilde{C}}_{i,ss}}{(1-a_{4}{\tilde{C}}_{C1,ss} -\frac{a_{4}b_{4}}{b_{5}}{\tilde{C}}_{VC \cdot C1,ss})^{2}b_{5}}. \end{aligned}$$

We added Eq. (26) to the right-hand side of Eq. (23), leading to the following matrix equation:

30 $$\begin{aligned}&\frac{\partial }{\partial {\tilde{t}}} \begin{pmatrix} \Delta {\tilde{C}}_{M2}\\ \Delta {\tilde{C}}_{VC}\\ \Delta {\tilde{C}}_{C1}\\ \Delta {\tilde{C}}_{VC \cdot C1} \end{pmatrix}= \begin{pmatrix} d_{1,M2} &{} 0 &{} d_{2,M2} &{} d_{3,M2}\\ 0 &{} d_{1,VC} &{} d_{2,VC} &{} d_{3,VC}\\ 0 &{} 0 &{} 0 &{} 0\\ 0 &{} 0 &{} 0 &{} 0\\ \end{pmatrix} \begin{pmatrix} \frac{\partial ^{2}\Delta {\tilde{C}}_{M2}}{\partial \tilde{x}^{2}}\\ \frac{\partial ^{2}\Delta {\tilde{C}}_{VC}}{\partial \tilde{x}^{2}}\\ \frac{\partial ^{2}\Delta {\tilde{C}}_{C1}}{\partial \tilde{x}^{2}}\\ \frac{\partial ^{2}\Delta {\tilde{C}}_{VC \cdot C1}}{\partial \tilde{x}^{2}} \end{pmatrix}\nonumber \\&\quad +\begin{pmatrix} -1 &{} {\tilde{C}}_{C1,ss} &{} {\tilde{C}}_{VC,ss} &{} 0\\ 0 &{} -1-b_{1}{\tilde{C}}_{C1,ss} &{} 1-b_{1}{\tilde{C}}_{VC,ss} &{} b_{1}\\ -b_{3} &{} -b_{4}{\tilde{C}}_{C1,ss} &{} -b_{4}{\tilde{C}}_{VC,ss} &{} b_{4}\\ 0 &{} b_{5}{\tilde{C}}_{C1,ss} &{} b_{5}{\tilde{C}}_{VC,ss} &{} -b_{5}\\ \end{pmatrix} \begin{pmatrix} \Delta {\tilde{C}}_{M2}\\ \Delta {\tilde{C}}_{VC}\\ \Delta {\tilde{C}}_{C1}\\ \Delta {\tilde{C}}_{VC \cdot C1} \end{pmatrix}. \end{aligned}$$

The spatial eigenvalue problem associated with Eq. (30) is satisfied by a Fourier series: $\sum \nolimits _{n \in {\mathbb {Z}}}\Big [{\varvec{c}}_{n}^{c}\cos (n\pi \tilde{x})+{\varvec{c}}_{n}^{s}\sin (n\pi \tilde{x})\Big ]$, where ${\varvec{c}}_{n}^{c}$’s and ${\varvec{c}}_{n}^{s}$’s are constant vectors. This solution must satisfy the linearised boundary conditions. At the HSS, $\left| a_{4}{\tilde{C}}_{C1}+\frac{a_{4}b_{4}}{b_{5}}{\tilde{C}}_{VC \cdot C1}\right| \ll 1$, so ${\tilde{C}}_{i}\big (1-a_{4}{\tilde{C}}_{C1}-\frac{a_{4}b_{4}}{b_{5}}{\tilde{C}}_{VC \cdot C1}\big )^{-1} \approx {\tilde{C}}_{i}\big (1+a_{4}{\tilde{C}}_{C1}+\frac{a_{4}b_{4}}{b_{5}}{\tilde{C}}_{VC \cdot C1}+\cdots \big )$. We applied this result to Eqs. (17) and (18) and ignored the nonlinear terms. As a result, $\frac{\partial {\tilde{C}}_{i}}{\partial \tilde{x}}=0$ where $\tilde{x}=0$ and $\tilde{x}=1$. Only the cosine terms satisfy the linearised boundary conditions, so the solution is $\sum \nolimits _{n \in {\mathbb {Z}}}{\varvec{c}}_{n}^{c}\cos (n\pi \tilde{x})$.

The solution of Eq. (30) is the product of the Fourier cosine series and an exponential term: $\sum \nolimits _{n \in {\mathbb {Z}}}{\varvec{c}}_{n}^{c}e^{\sigma _{n}{\tilde{t}}}\cos (n\pi \tilde{x})$, where $\sigma _{n}$ is an eigenvalue associated with the $n^{\mathrm{th}}$ wavenumber. We can abbreviate the solution by defining that $\varvec{w}_{n}={\varvec{c}}_{n}^{c}\cos (n\pi \tilde{x})$. Each instance of $\varvec{w}_{n}$ is a Fourier mode with the wavenumber: $k=n\pi$.

We substituted the solution into Eq. (30), leading to the following:

31 $$\begin{aligned} \big (B-\sigma _{n} I\big ){\varvec{w}}_{n}=0. \end{aligned}$$

*B* is a square matrix:

32 $$\begin{aligned} B=\begin{pmatrix} -1-k^{2}d_{1,M2} &{}\quad {\tilde{C}}_{C1,ss} &{}\quad {\tilde{C}}_{VC,ss}-k^{2}d_{2,M2} &{}\quad -k^{2}d_{3,M2}\\ 0 &{}\quad -1-b_{1}{\tilde{C}}_{C1,ss}-k^{2}d_{1,VC} &{}\quad 1-b_{1}{\tilde{C}}_{VC,ss}-k^{2}d_{2,VC} &{}\quad b_{1}-k^{2}d_{3,VC}\\ -b_{3} &{}\quad -b_{4}{\tilde{C}}_{C1,ss} &{} -b_{4}{\tilde{C}}_{VC,ss} &{}\quad b_{4}\\ 0 &{}\quad b_{5}{\tilde{C}}_{C1,ss} &{}\quad b_{5}{\tilde{C}}_{VC,ss} &{}\quad -b_{5}\\ \end{pmatrix}. \end{aligned}$$

If the solution of Eq. (30) is nontrivial, $$\begin{vmatrix} B-\sigma _{n} I\end{vmatrix} =0$$ for each value of *n* and hence *k*. It is a quartic equation for $\sigma _{n}$, the eigenvalue of *B*; its four solutions give $\sigma _{n}$ in terms of $k^{2}$. At each *k* value, if at least one of the four eigenvalues has a positive real part, that Fourier mode grows with time, making it an unstable noise component for the HSS. Because the eigenvalues are expressed in terms of $n^{2}$, negative values of *n* are redundant and trivial.

To conclude, the fourth constraint on the Turing space is as follows. At least one eigenvalue of *B* must have a positive real part at at least one *k* value.

### Dispersion Relation

At each point in the parametric space, the four eigenvalues of the matrix *B* vary with *k* ($k=n\pi$). At each value of *k*, the real part of one eigenvalue is larger than or equal to the other three. The relation between this maximum real part, $Re(\sigma _{n, \max })$, and *k* is the dispersion relation for that point in the parametric space.

If Turing’s mechanism works at that point in the parametric space, the dispersion relation must peak in a relevant range of finite Fourier modes. The Fourier mode at the peak grows faster than the other noise components, leading to a periodic pattern with a wavelength of $\frac{2\pi }{k}$. The nondimensionalised length of our domain is 1, so a visible pattern must have a smaller wavelength. On the other hand, the LEC diameter is 10 µm or 0.023 after nondimensionalisation, so a pattern with a smaller wavelength cannot be resolved by an LEC.

The fifth constraint on the Turing space follows from the reasoning in the previous paragraph, i.e. the maximum of a dispersion relation must be in the relevant range of Fourier modes, $0.023<\frac{2\pi }{k}<1$. This constraint ensures that the resulting pattern is visible in the zebrafish embryo and can be resolved by an LEC.

In general, if Turing’s mechanism works, only a finite number of Fourier modes can be unstable. Otherwise, the result will be noises rather than a pattern. The constraints we have discussed so far ensure that a qualifying dispersion relation is negative when $n=0$ and turns positive to reach its peak. To ensure a finite band of unstable Fourier modes in a qualifying dispersion relation, we now stipulate that the dispersion relation must turn negative again after its peak.

The sixth constraint on the Turing space is therefore as follows: a dispersion relation must cross the x-axis to the right of its maximum.

### Summary

The Turing space of our model is the region of its parametric space where Turing’s mechanism is expected to work. Each point in the Turing space is called a Turing point in this paper. Integrating our findings in this section, we summarise below the constraints which must be satisfied by a Turing point.

1. $C_{C1,ss}>1\times 10^{-4}$ M. This inequality ensures that there is sufficient collagen I to justify our model assumptions. In particular, it makes sure the HSS is in the diffusion-dominant regime.
2. $b_{2}<b_{3}$. This inequality ensures the HSS concentrations are scaled properly, i.e. the nondimensionalised concentrations stay between 0 and 1.
3. The eigenvalues of *A* must have negative real parts to ensure that the HSS is stable in response to homogeneous perturbations.
4. At least one of the eigenvalues of *B* must have a positive real part at at least one *k* value. This constraint ensures that the HSS is unstable in response to heterogeneous perturbations.
5. The dispersion relation of the Turing point peaks in the relevant range of Fourier modes ($2<n<87$). It means one Fourier mode dominates the other and its wavelength is smaller than the domain size, but larger than the LEC diameter.
6. The dispersion relation must turn negative after reaching the maximum. As a result, only a finite number of Fourier modes are unstable.

## Turing Space

We applied the results of our linear stability analysis to find the Turing space. In this section, we describe the steps we took and the outcome we obtained.

### Turing Point Candidates

Our model is analytically intractable. We circumvented the problem by exploring the parametric space one point at a time. Before we did so, we had to come up with a sample of parametric combinations for screening.

The parametric combination summarised in Table 3 is based on experimental results, so the region of the parametric space around this reference point is physically relevant. On this basis, we decided to sample in the vicinity of the reference point.

We were dealing with an eight-dimensional problem. The five kinetic parameters, $b_{i}$’s, can be varied independently; $a_{1,j}$’s and $a_{2,j}$’s depend on three-dimensional parameters, $D^{\infty }_{M2}$, $D^{\infty }_{VC}$, and $\mu$, only; $a_{3}$ and $a_{4}$ are fixed. In total, there are eight independent dimensions in which variations are possible. We sampled in all eight dimensions combinatorially.

Finally, we had to decide on the sample size. To ensure a broad coverage, we considered five orders of magnitude in each dimension. To save computational cost, we sampled on a logarithmic scale.

For example, we considered $b_{1}$ at $1.90\times 10^{3}$, $1.90\times 10^{4}$, $1.90\times 10^{5}$ (reference point), $1.90\times 10^{6}$, and $1.90\times 10^{7}$. In the sample we obtained, each of these $b_{1}$ values is paired with every permissible combination of the remaining seven parameters, each of which has five permissible values.

Following these steps, we generated a sample of $5^{9}$ or 1,953,125 parametric combinations, which will be called Turing point candidates henceforth. We did not generate a larger sample because of the higher computational cost.

### Screening for Turing Points

Using a Python program, we tested each candidate against the six constraints summarised in Sect. 3.5. The aim was to screen the candidates for Turing points. However, when we consider the results, we must be wary of our method’s limitations.

First, due to the analytically intractable nature of our model, we could only assess a finite number of Fourier modes. We assessed *n* in the range of 0–87. Therefore, we missed any dispersion relations which are negative in the assessed range, but which turn positive beyond it. However, as discussed in Sect. 3, the Fourier modes beyond this range are not relevant to the present problem anyway because their wavelengths are smaller than an LEC.

Second, even if a dispersion relation is positive in parts of the assessed range, it may peak beyond the range. The predicted wavelength at such a Turing point is wrong.

Third, a dispersion relation may peak, drop to a negative value, and then turn positive again. This type of dispersion relation may have an infinite number of unstable Fourier modes.

Fourth, a dispersion relation may decrease from its maximum and cross the x-axis outside the assessed range. This case is similar to the first one and such a dispersion relation is a false negative, but the Fourier modes beyond the assessed range are not relevant to the present problem.

Fifth, there may be two or more identical maxima in a dispersion relation. Multiple wavelengths will be predicted at such a Turing point.

### Parametric Distributions

Out of the 1,953,125 Turing point candidates, we have found 94 Turing points: a sample of the Turing space. On this basis, we argue that VEGFC *can* form Turing patterns in the zebrafish embryo. More precisely, we mean its functional relations with MMP2 and collagen I support Turing pattern formation; they are wired in the right way.

Whether VEGFC *does* form Turing patterns is another question and one dependent on the physiological conditions. Fewer than 0.01% of the candidates are Turing points, so the embryo needs a control mechanism to create the conditions at the Turing points. For example, the zebrafish may be genetically programmed to produce VEGFC and MMP2 at specific rates in the time frame of interest. We now turn our attention to what this control mechanism, if it exists, entails.

**Fig. 4:**
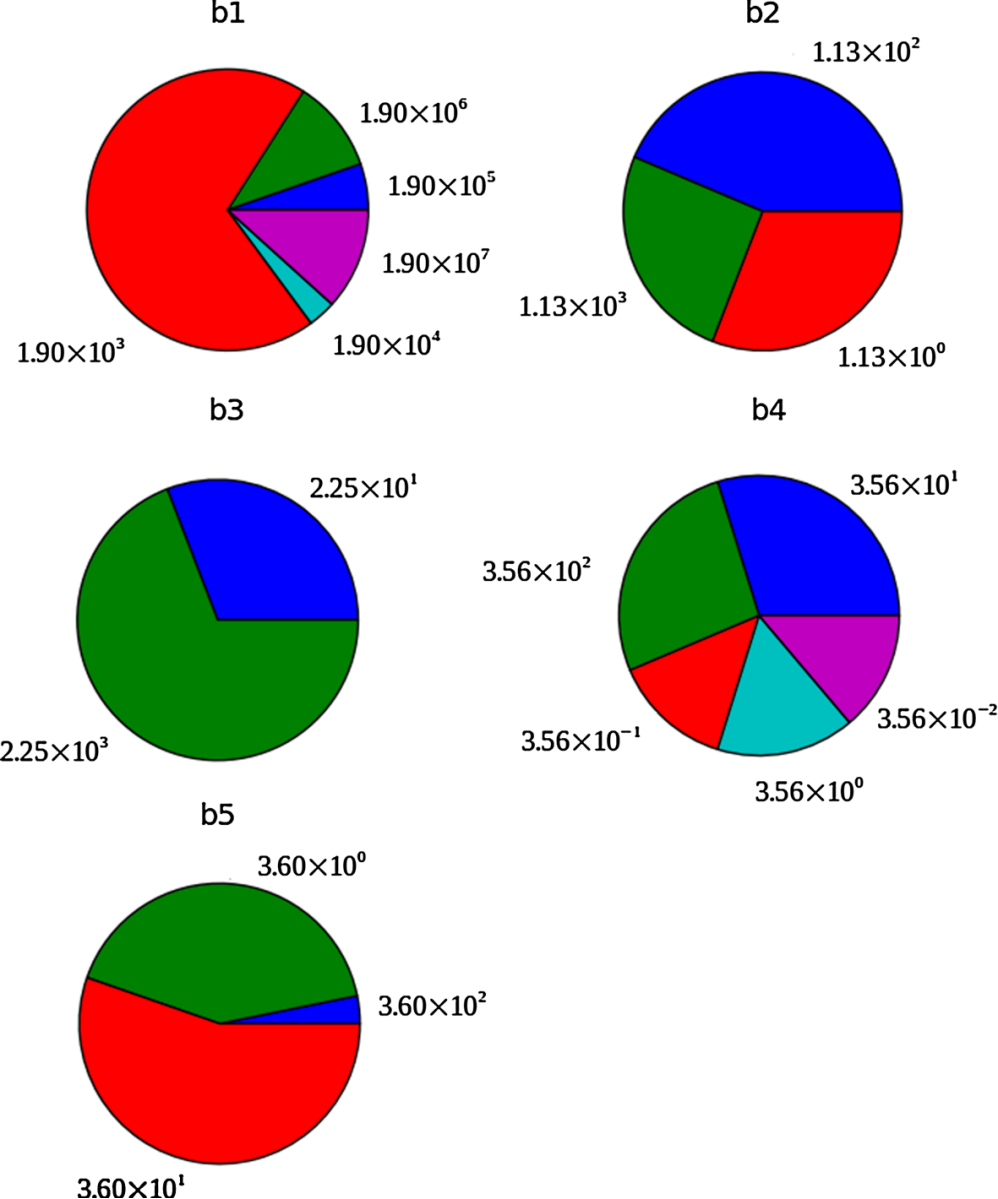
Parametric distributions in a sample of 94 Turing points. The parameters are defined as follows: $b_{1}=k^{on}_{VC,C1}\tau C_{C1,s}$, $b_{2}=\frac{P_{C1}\tau }{C_{C1,s}}$, $b_{3}=\frac{k^{cat}_{M2,C1}\tau P_{M2}}{k^{deg}_{M2}C_{C1,s}}$, $b_{4}=\frac{k^{on}_{VC,C1}\tau P_{VC}}{k^{deg}_{VC}}$, and $b_{5}=k^{off}_{VC,C1}\tau$

We cannot draw definitive conclusions from a finite number of Turing points, but they offer insights into the Turing space. Figure 4 shows the parametric distributions in this sample. The transport parameters are not shown because they are not independent of each other; no insights can be gained from their independent distributions.

Small values of $b_{1}$ are common in the sample, suggesting a smaller-than-estimated $k^{on}_{VC,C1}$ favours Turing’s mechanism. The $b_{2}$ distribution is biased towards the two largest values, suggesting a larger-than-estimated $P_{C1}$ favours Turing’s mechanism. The $b_{3}$ distribution is dominated by the largest value, suggesting a higher-than-estimated $k^{cat}_{M2,C1}$ or $P_{M2}$ is favourable. All five values of $b_{4}$ are evenly represented in the sample; $b_{4}$ is controlled by $k^{on}_{VC,C1}$ and $P_{VC}$, while a smaller-than-estimated $k^{on}_{VC,C1}$ seems to favour Turing’s mechanism, so the mechanism seems to be insensitive to $P_{VC}$. The $b_{5}$ distribution is dominated by the reference value and contains its two adjacent values, suggesting that the estimated $k^{off}_{VC,C1}$ is favourable for Turing’s mechanism.

Figure 4 tells us nothing about the correlations between the five kinetic parameters. For example, if a Turing point has a $b_{1}$ value of 1900, its $b_{3}$ may always be 22.5. Furthermore, we are still ignorant of how the form of a dispersion relation varies in the Turing space. We will discuss the final issue in the next subsection.

### Dispersion Relations

Our 94 Turing points lie at different distances from the reference point. The reference point has its basis in the literature, so the Turing points closest to it deserve special attention.

Let us label the distance between a Turing point and the reference point by *S*. Let us also label the parametric values at the reference point by $a^{ref}$’s and $b^{ref}$’s. Using these notations, we can write down the equation for the relative distance between a Turing point and the reference point:

33 $$\begin{aligned} S^{2}= & {} \left( \frac{b_{1}-b_{1}^{ref}}{b_{1}^{ref}}\right) ^{2} +\left( \frac{b_{2}-b_{2}^{ref}}{b_{2}^{ref}}\right) ^{2} +\left( \frac{b_{3}-b_{3}^{ref}}{b_{3}^{ref}}\right) ^{2} +\left( \frac{b_{4}-b_{4}^{ref}}{b_{4}^{ref}}\right) ^{2}\nonumber \\&+\,\left( \frac{b_{5}-b_{5}^{ref}}{b_{5}^{ref}}\right) ^{2} +\left( \frac{a_{1,M2}-a_{1,M2}^{ref}}{a_{1,M2}^{ref}} \right) ^{2}+\left( \frac{a_{1,VC}-a_{1,VC}^{ref}}{a_{1,VC}^{ref}}\right) ^{2}\nonumber \\&+\,\left( \frac{a_{2,M2}-a_{2,M2}^{ref}}{a_{2,M2}^{ref}} \right) ^{2}+\left( \frac{a_{2,VC}-a_{2,VC}^{ref}}{a_{2,VC}^{ref}}\right) ^{2}. \end{aligned}$$

We calculated *S* for each of the 94 Turing points and selected the ten points with the smallest *S* values. The results are given in Table 5.

**Table 5:** The ten Turing points closest to the reference point in the parametric space

$b_{1}$	$b_{2}$	$b_{3}$	$b_{4}$	$b_{5}$	$a_{1,M2}$	$a_{1,VC}$	$a_{2,M2}$	$a_{2,VC}$	*S*	$\lambda$
$1.9\times 10^{6}$	1.13	22.5	35.6	3.6	0.0451	2.66	107	1.81	99.83	0.400
$1.9\times 10^{6}$	1.13	22.5	35.6	3.6	0.451	2.66	107	18.1	100.23	0.038
$1.9\times 10^{6}$	1.13	22.5	35.6	3.6	4.51	26.6	107	18.1	100.63	0.125
$1.9\times 10^{5}$	1.13	22.5	356	36	0.451	2.66	107	18.1	140.30	0.111
$1.9\times 10^{6}$	1.13	22.5	356	36	0.0451	2.66	107	1.81	140.30	0.400
$1.9\times 10^{4}$	1.13	22.5	356	36	0.451	2.66	107	18.1	140.30	0.400
$1.9\times 10^{7}$	1.13	22.5	35.6	3.6	0.0451	2.66	107	1.81	140.31	0.133
$1.9\times 10^{4}$	1.13	22.5	356	36	0.0451	0.266	107	18.1	140.31	0.125
$1.9\times 10^{5}$	1.13	22.5	356	36	4.51	26.6	107	18.1	140.59	0.333
$1.9\times 10^{3}$	113	2250	3.56	36	0.451	2.66	107	18.1	140.59	0.250

The parameters are defined as follows: $b_{1}=k^{on}_{VC,C1}\tau C_{C1,s}$, $b_{2}=\frac{P_{C1}\tau }{C_{C1,s}}$, $b_{3}=\frac{k^{cat}_{M2,C1}\tau P_{M2}}{k^{deg}_{M2}C_{C1,s}}$, $b_{4}=\frac{k^{on}_{VC,C1}\tau P_{VC}}{k^{deg}_{VC}}$, $b_{5}=k^{off}_{VC,C1}\tau$, $a_{1,M2}=\frac{D^{\infty }_{M2}\tau }{L^{2}}$, $a_{1,VC}=\frac{D^{\infty }_{VC}\tau }{L^{2}}$, $a_{2,M2}=\frac{k_{B}T}{6\pi \mu D^{\infty }_{M2} r_{f}}$, and $a_{2,VC}=\frac{k_{B}T}{6\pi \mu D^{\infty }_{VC} r_{f}}$. *S* is the distance between a Turing point and the reference point. The last column gives the wavelength ($\lambda$) of the fastest growing noise component (Fourier mode) at each Turing point

The first commonality of these ten Turing points is that VEGFC always diffuses faster than MMP2.

**Fig. 5:**
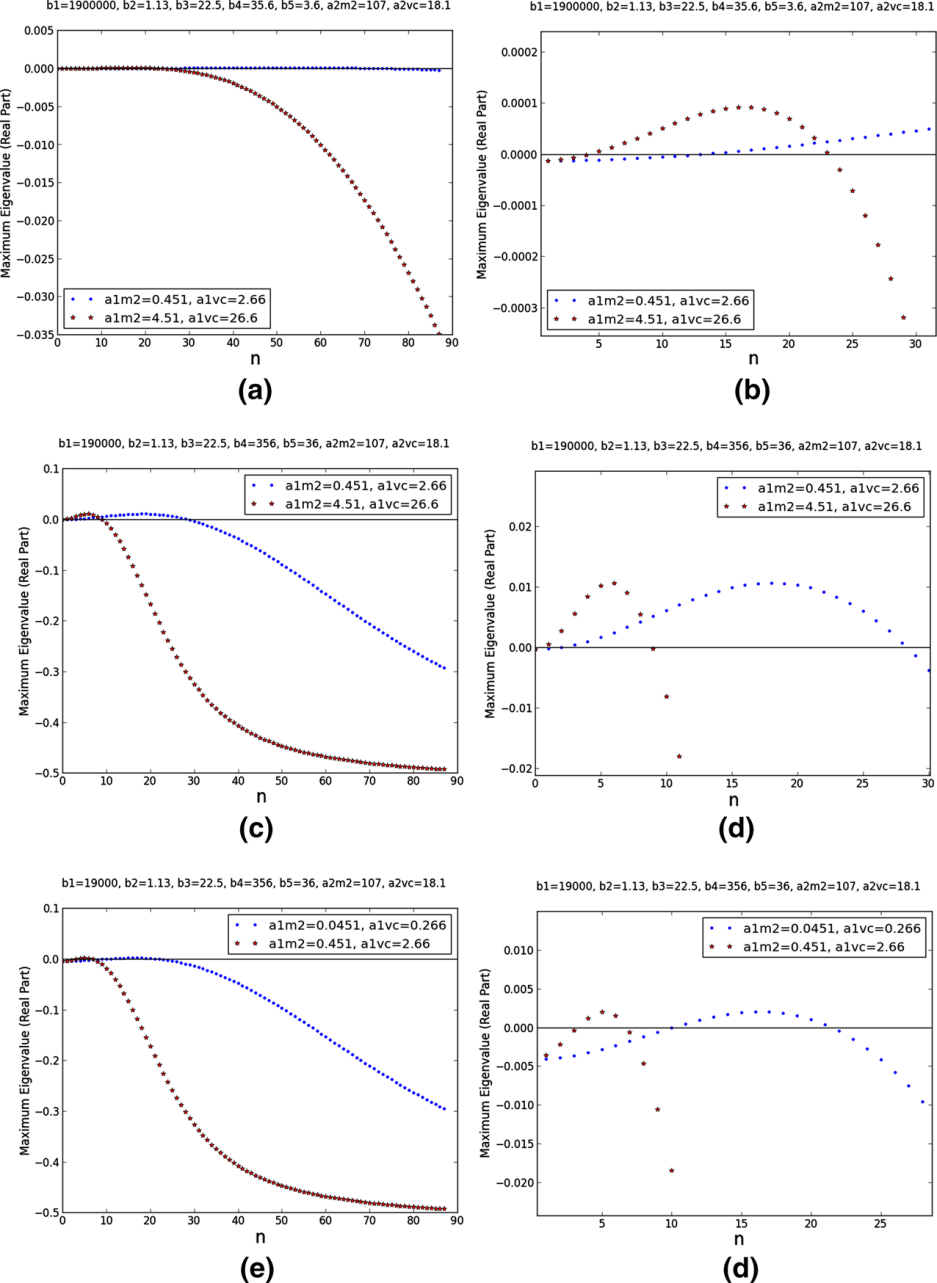
Comparison plots of the dispersion relations at selected Turing points. They are among the ten Turing points closest to the reference point ($b_{1}=1.90\times 10^{5}$, $b_{2}=1.13\times 10^{1}$, $b_{3}=2.25\times 10^{1}$, $b_{4}=3.56$, $b_{5}=3.60\times 10^{1}$, $a_{1,M2}=4.51$, $a_{1,VC}=2.66$, $a_{2,M2}=1.07$, and $a_{2,VC}=1.81$). The plots on the right are magnified versions of their counterparts on the left. On the *x*-axes, *n* is the integer in the wavenumber ($k=n\pi$). For **a** and **b**, $b_{1}=1.9\times 10^{6}$, $b_{2}=1.13$, $b_{3}=22.5$, $b_{4}=35.6$, $b_{5}=3.6$, $a_{2,M2}=107$, and $a_{2,VC}=18.1$; for the blue trend and small dots, $a_{1,M2}=0.451$ and $a_{1,VC}=2.66$; for the red trend and big dots, $a_{1,M2}=4.51$ and $a_{1,VC}=26.6$. For **c** and **d**, $b_{1}=1.9\times 10^{5}$, $b_{2}=1.13$, $b_{3}=22.5$, $b_{4}=356$, $b_{5}=36$, $a_{2,M2}=107$, and $a_{2,VC}=18.1$; for the blue trend and small dots, $a_{1,M2}=0.451$ and $a_{1,VC}=2.66$; for the red trend and big dots, $a_{1,M2}=4.51$ and $a_{1,VC}=26.6$. For **e** and **f**, $b_{1}=1.9\times 10^{4}$, $b_{2}=1.13$, $b_{3}=22.5$, $b_{4}=356$, $b_{5}=36$, $a_{2,M2}=107$, and $a_{2,VC}=18.1$; for the blue trend and small dots, $a_{1,M2}=0.0451$ and $a_{1,VC}=0.266$; for the red trend and big dots, $a_{1,M2}=0.451$ and $a_{1,VC}=2.66$ (Color figure online)

The second feature is illustrated in Fig. 5. It shows the comparison between three pairs of dispersion relations from Table 5: 2 and 3, 4 and 9, and 6 and 8. In each pair, only the $a_{1,j}$’s differ. The $a_{2,j}$’s determine any volume exclusion effects. It follows that in each selected pair, only the diffusion rates in pure interstitial fluid change. According to Fig. 5, faster diffusion leads to a more ‘compressed’ dispersion relation. Physically, it means fewer unstable noise components and these components have longer wavelengths. Biologically, faster diffusion is favourable for Turing’s mechanism because when fewer noise components are unstable, the pattern is more regular.

**Fig. 6:**
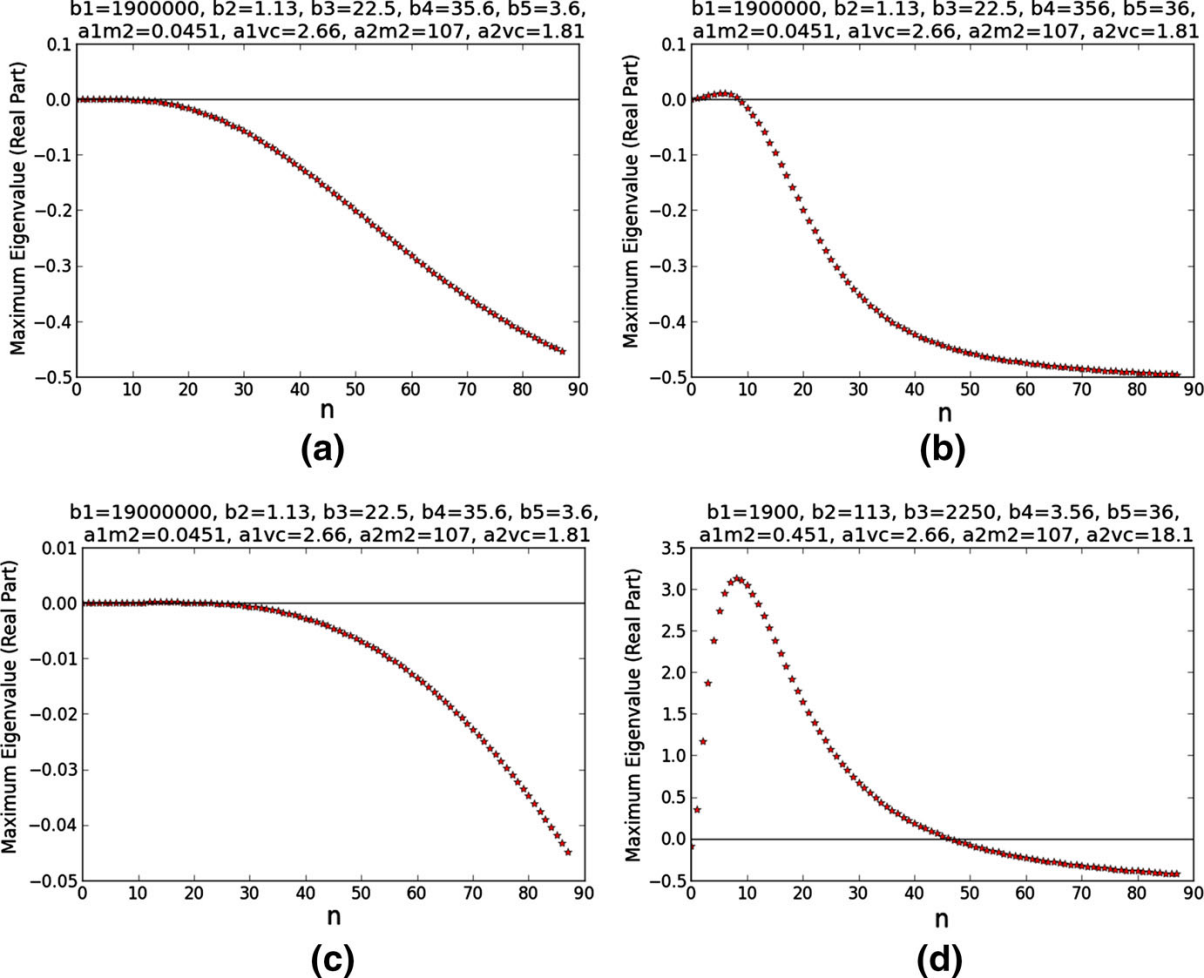
Dispersion relations at selected Turing points. They are among the ten Turing points closest to the reference point ($b_{1}=1.90\times 10^{5}$, $b_{2}=1.13\times 10^{1}$, $b_{3}=2.25\times 10^{1}$, $b_{4}=3.56$, $b_{5}=3.60\times 10^{1}$, $a_{1,M2}=4.51$, $a_{1,VC}=2.66$, $a_{2,M2}=1.07$, and $a_{2,VC}=1.81$). On the *x*-axes, *n* is the integer in the wavenumber ($k=n\pi$). For **a**, $b_{1}=1.9\times 10^{6}$, $b_{2}=1.13$, $b_{3}=22.5$, $b_{4}=35.6$, $b_{5}=3.6$, $a_{1,M2}=0.0451$, $a_{1,VC}=2.66$, $a_{2,M2}=107$, and $a_{2,VC}=1.81$. For **b**, $b_{1}=1.9\times 10^{6}$, $b_{2}=1.13$, $b_{3}=22.5$, $b_{4}=356$, $b_{5}=36$, $a_{1,M2}=0.0451$, $a_{1,VC}=2.66$, $a_{2,M2}=107$, and $a_{2,VC}=1.81$. For **c**, $b_{1}=1.9\times 10^{7}$, $b_{2}=1.13$, $b_{3}=22.5$, $b_{4}=35.6$, $b_{5}=3.6$, $a_{1,M2}=0.0451$, $a_{1,VC}=2.66$, $a_{2,M2}=107$, and $a_{2,VC}=1.81$. For **d**, $b_{1}=1.9\times 10^{3}$, $b_{2}=113$, $b_{3}=2250$, $b_{4}=3.56$, $b_{5}=36$, $a_{1,M2}=0.451$, $a_{1,VC}=2.66$, $a_{2,M2}=107$, and $a_{2,VC}=18.1$

The dispersion relations at the remaining Turing points in Table 5 are plotted in Fig. 6.

### Bifurcation

When we discussed the model parameters, we commented on the uncertainty of $P_{M2}$ and $P_{VC}$. Since they control $b_{3}$ and $b_{4}$, respectively, we perturbed $b_{3}$ and $b_{4}$ independently. In other words, they were used as bifurcation parameters.

We had to pick a reference Turing point for our bifurcation analysis. The dispersion relations in the last subsection are numerical results, so they contain rounding errors. In a dispersion relation like the one in Fig. 6a, the smallness of the eigenvalues means they are highly sensitive to these errors; the predicted dominant Fourier mode may thus be inaccurate. By contrast, the dispersion relation in Fig. 6d has the sharpest peak out of the ten dispersion relations plotted in Figs. 5 and 6. The large eigenvalues mean the trends shown by this dispersion relation are likely to be less sensitive to rounding errors. Therefore, we performed our bifurcation analysis around its corresponding Turing point, the final one in Table 5.

**Fig. 7:**
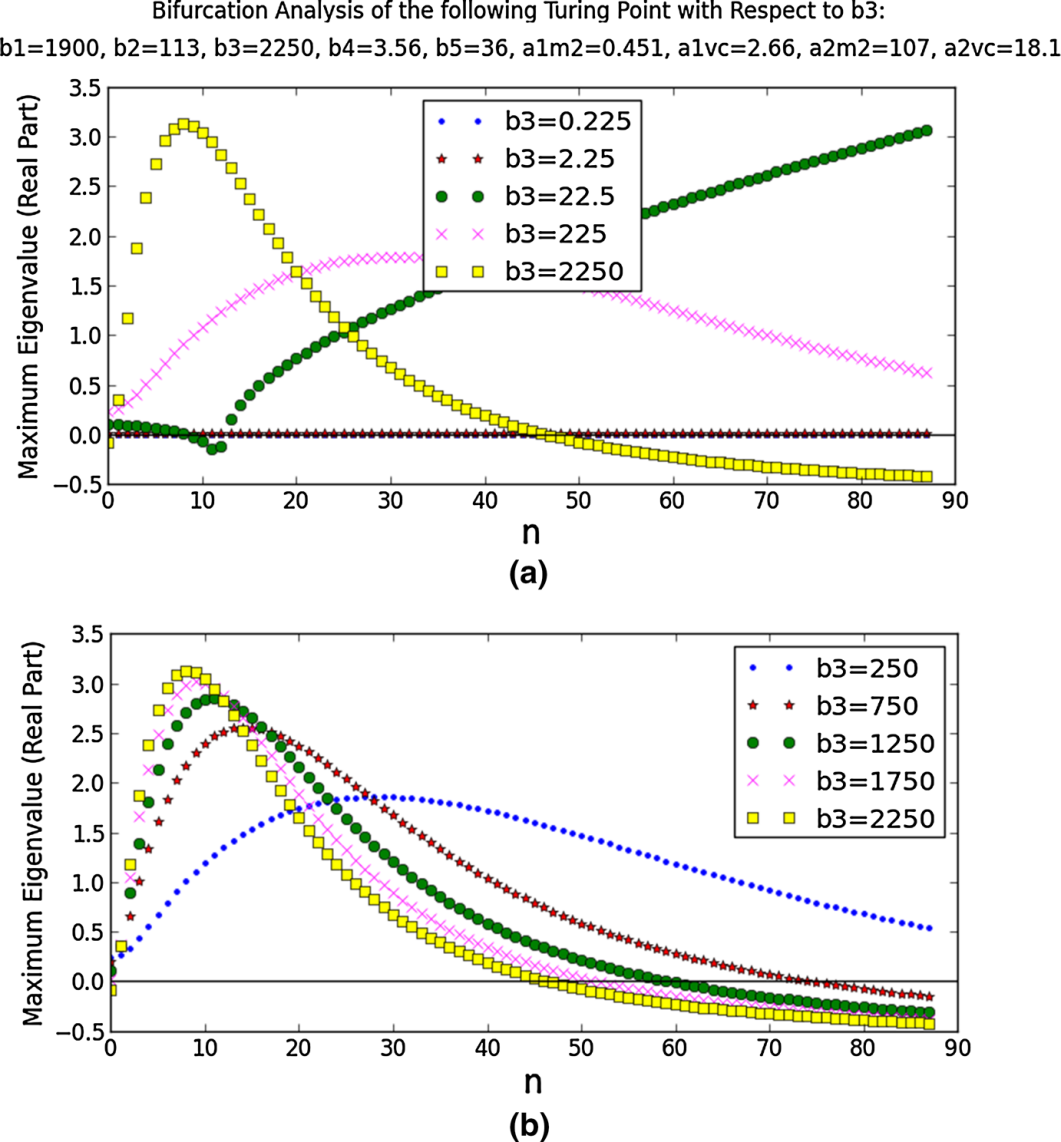
Bifurcation analysis of a selected Turing point with respect to $b_{3}$. It is one of the ten Turing points closest to the reference point ($b_{1}=1.90\times 10^{5}$, $b_{2}=1.13\times 10^{1}$, $b_{3}=2.25\times 10^{1}$, $b_{4}=3.56$, $b_{5}=3.60\times 10^{1}$, $a_{1,M2}=4.51$, $a_{1,VC}=2.66$, $a_{2,M2}=1.07$, and $a_{2,VC}=1.81$). At this Turing point, $b_{1}=1.9\times 10^{3}$, $b_{2}=113$, $b_{3}=\frac{k^{cat}_{M2,C1}\tau P_{M2}}{k^{deg}_{M2}C_{C1,s}}=2250$, $b_{4}=3.56$, $b_{5}=36$, $a_{1,M2}=0.451$, $a_{1,VC}=2.66$, $a_{2,M2}=107$, and $a_{2,VC}=18.1$. On the *x*-axes, *n* is the integer in the wavenumber ($k=n\pi$). **a** considers a wider range of $b_{3}$ than **b**

Figure 7 shows the dispersion relations at different $b_{3}$ values. In Fig. 7a, the trends are inconsistent, so our only conclusion is that $b_{3}$ is a very sensitive parameter. We note that $b_{3}=\frac{k^{cat}_{M2,C1}\tau P_{M2}}{k^{deg}_{M2}C_{C1,s}}$. Because $k^{cat}_{M2,C1}$ and $P_{M2}$ both parametrise the MMP2-catalysed degradation of collagen I, it may explain the sensitivity of $b_{3}$: its change affects both MMP2 production and action. Out of the five dispersion relations in Fig. 7a, only the one with the largest $b_{3}$ meets all the criteria of a Turing point. When we noticed this result, we repeated the bifurcation analysis around it, considering a narrower range of $b_{3}$. Figure 7b shows the results. An increase in $b_{3}$ leads to a smaller eigenvalue when $n=0$, stabilising the HSS with respect to homogeneous perturbations; it also leads to fewer unstable Fourier modes (hence a more regular spatial pattern) and these modes have smaller wavenumbers (longer wavelengths).

**Fig. 8:**
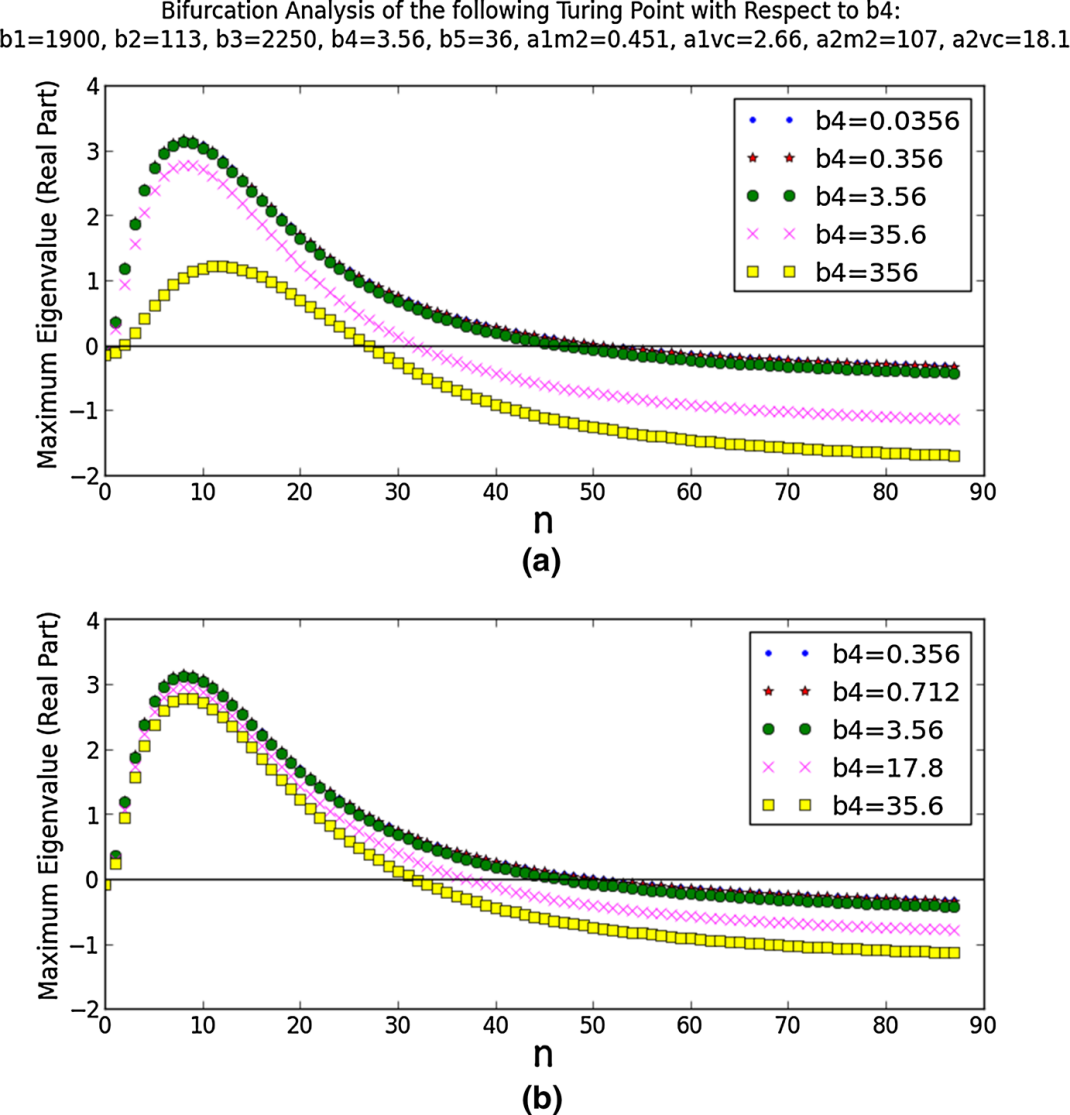
Bifurcation analysis of a selected Turing point with respect to $b_{4}$. It is one of the ten Turing points closest to the reference point ($b_{1}=1.90\times 10^{5}$, $b_{2}=1.13\times 10^{1}$, $b_{3}=2.25\times 10^{1}$, $b_{4}=3.56$, $b_{5}=3.60\times 10^{1}$, $a_{1,M2}=4.51$, $a_{1,VC}=2.66$, $a_{2,M2}=1.07$, and $a_{2,VC}=1.81$). At this Turing point, $b_{1}=1.9\times 10^{3}$, $b_{2}=113$, $b_{3}=2250$, $b_{4}=\frac{k^{on}_{VC,C1}\tau P_{VC}}{k^{deg}_{VC}}=3.56$, $b_{5}=36$, $a_{1,M2}=0.451$, $a_{1,VC}=2.66$, $a_{2,M2}=107$, and $a_{2,VC}=18.1$. On the *x*-axes, *n* is the integer in the wavenumber ($k=n\pi$). **a** considers a wider range of $b_{4}$ than **b**

Figure 8 shows the dispersion relations at different $b_{4}$ values. They all satisfy the six criteria, so the HSS is less sensitive to $b_{4}$ than $b_{3}$. The trends are opposite to those in Fig. 7: a decrease in $b_{4}$ results in fewer unstable Fourier modes and these modes have smaller wavenumbers (longer wavelengths).

### Patterning Mechanism

So far in this section, we have considered the structure of an unchanging Turing space. Before we summarise our findings, we will take a step back, analyse the patterning mechanism itself, and explain how the Turing space changes if the patterning mechanism is tweaked.

At the beginning of Sect. 3, we discussed the three differences between our model and the classic instance of Turing’s mechanism (Gierer and Meinhardt 1972). First, VEGFC and MMP2 do not form a self-activator-self-inhibitor pair. Specifically, VEGFC does not stimulate its own production and MMP2 does not inhibit VEGFC production. Second, VEGFC binds to an immobile substrate (collagen I) reversibly.

After examining Fig. 3, we came to a conclusion. The second and third differences may cancel each other out, reducing our model to the classic instance. The positive feedback loop involving VEGFC, collagen I, and VEGFC-bound collagen I allows VEGFC to stimulate its own production. The negative feedback loop involving VEGFC, MMP2, and collagen I allows MMP2 to inhibit VEGFC production by degrading collagen I, thus shutting down the positive feedback loop.

To test our hypothesis, we switched off the linearised interaction between VEGFC and MMP2, leading to new *A* and *B*:

34 $$\begin{aligned} A= & {} \begin{pmatrix} -1 &{} 0 &{} {\tilde{C}}_{VC,ss} &{} 0\\ 0 &{} -1-b_{1}{\tilde{C}}_{C1,ss} &{} 1-b_{1}{\tilde{C}}_{VC,ss} &{} b_{1}\\ -b_{3} &{} -b_{4}{\tilde{C}}_{C1,ss} &{} -b_{4}{\tilde{C}}_{VC,ss} &{} b_{4}\\ 0 &{} b_{5}{\tilde{C}}_{C1,ss} &{} b_{5}{\tilde{C}}_{VC,ss} &{} -b_{5}\\ \end{pmatrix}\; \text {and} \end{aligned}$$

35 $$\begin{aligned} B= & {} \begin{pmatrix} -1-k^{2}d_{1,M2} &{} 0 &{} {\tilde{C}}_{VC,ss}-k^{2}d_{2,M2} &{} -k^{2}d_{3,M2}\\ 0 &{} -1-b_{1}{\tilde{C}}_{C1,ss}-k^{2}d_{1,VC} &{} 1-b_{1}{\tilde{C}}_{VC,ss}-k^{2}d_{2,VC} &{} b_{1}-k^{2}d_{3,VC}\\ -b_{3} &{} -b_{4}{\tilde{C}}_{C1,ss} &{} -b_{4}{\tilde{C}}_{VC,ss} &{} b_{4}\\ 0 &{} b_{5}{\tilde{C}}_{C1,ss} &{} b_{5}{\tilde{C}}_{VC,ss} &{} -b_{5}\\ \end{pmatrix}.\nonumber \\ \end{aligned}$$

We repeated the screening procedure using Eqs. (34) and (35). We failed to find any Turing points among the same 1,953,125 candidates. The Turing space’s disappearance implies that the negative feedback loop is indispensable for the patterning mechanism.

Then, we turned to the positive feedback loop. We set $b_{1}$, $b_{4}$, and $b_{5}$ to zero before repeating the screening test. Once again, we failed to find any Turing points among the same 1,953,125 candidates. The positive feedback loop is indispensable for the patterning mechanism too.

The third difference between our model and the classic instance lies in volume exclusion, a feature not in the classic instance. In our model, collagen I takes away space wherein VEGFC and MMP2 can diffuse. This general property affects both mobile species, so it does not change their relative diffusion rates. We suspected that it may not contribute to the proposed patterning mechanism. Therefore, we set $d_{2,i}$ and $d_{3,i}$ to zero in Eqs. (28) and (29) before repeating the screening procedure. This time, we found 184 Turing points among the same 1,953,125 candidates; 88 of them are among the 94 Turing points found after the original screening. That we found a larger Turing space after switching off volume exclusion means volume exclusion is a thorn in the patterning mechanism.

### Summary

We now know that VEGFC *can* form Turing patterns due to its functional relations with MMP2 and collagen I. The responsible patterning mechanism has two indispensable components. First, reversible binding between VEGFC and collagen I leads to a positive feedback loop for VEGFC production. Second, VEGFC stimulates the production of MMP2, which degrades collagen I to shut down the first component. We also know that some modelled relations are not important to this patterning mechanism. For example, volume exclusion by collagen I actually makes the mechanism less potent.

However, because only 94 of the 1,953,125 candidates are Turing points, the zebrafish embryo needs a control mechanism to create the physiological conditions where VEGFC *does* form Turing patterns. Based on the sample of 94 Turing points, we obtained new insights into the Turing space of our model, insights pointing to what the control mechanism must accomplish. They are summarised below. It is important to note that they are relative to the reference point defined in Table 3. Furthermore, they are based on a small sample, so they are only preliminary results about the Turing space, not definitive conclusions.

1. Weak VEGFC–collagen I binding favours Turing’s mechanism.
2. A high turnover of collagen I favours Turing’s mechanism. Collagen I degradation by MMP2 stabilises the HSS with respect to homogeneous perturbations; it also reduces the number of unstable noise components.
3. Turing’s mechanism is not very sensitive to the production rate of VEGFC. However, a low production rate means fewer unstable noise components.
4. Fast diffusion of VEGFC and MMP2 favours Turing’s mechanism by creating fewer unstable noise components. A high diffusion rate of VEGFC relative to that of MMP2 also favours Turing’s mechanism.

## Computer Simulations

As discussed in the previous section, we predicted the existence of a Turing space for our model. In order to support our claim, we simulated the formation of VEGFC patterns in the trunk. In particular, the method used to find the Turing space has several limitations as explained earlier. If a Turing point is a false positive due to these limitations, it can be spotted in the simulation results. We were looking for an agreement between the predicted and simulated wavelengths at a Turing point.

**Fig. 9:**
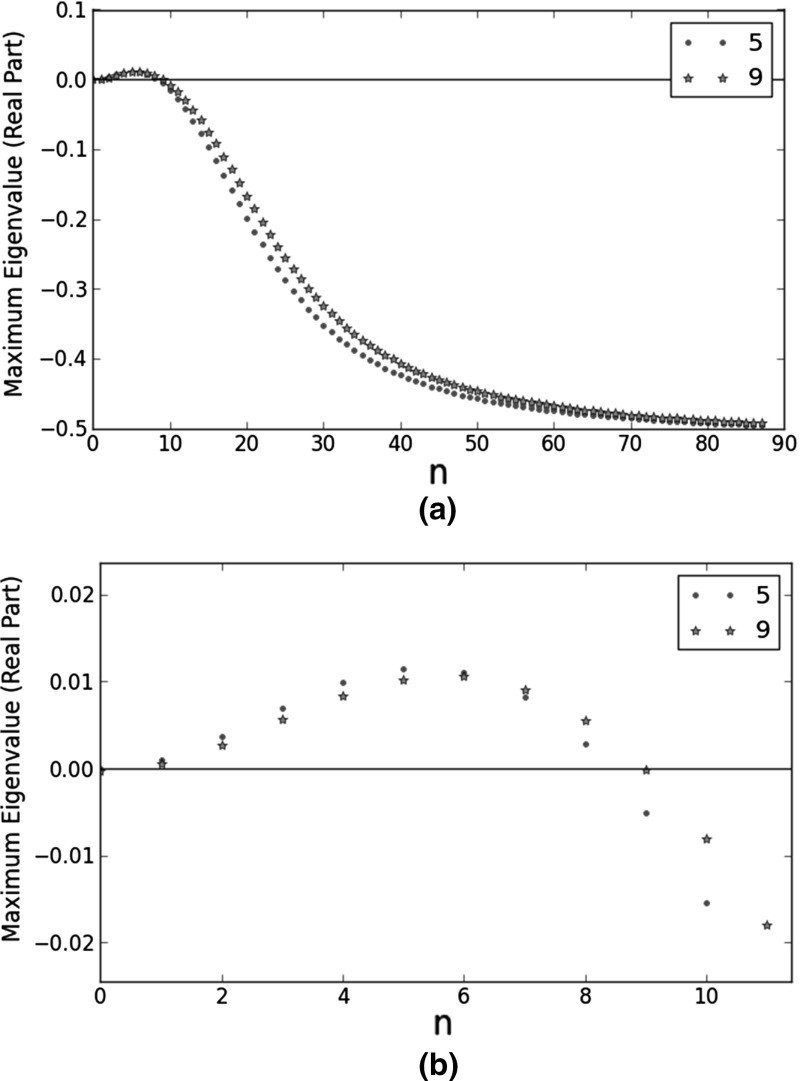
Dispersion relations at two Turing points. They are among the ten Turing points closest to the reference point ($b_{1}=1.90\times 10^{5}$, $b_{2}=1.13\times 10^{1}$, $b_{3}=2.25\times 10^{1}$, $b_{4}=3.56$, $b_{5}=3.60\times 10^{1}$, $a_{1,M2}=4.51$, $a_{1,VC}=2.66$, $a_{2,M2}=1.07$, and $a_{2,VC}=1.81$). At point 5, $b_{1}=1.9\times 10^{6}$, $b_{2}=1.13$, $b_{3}=22.5$, $b_{4}=356$, $b_{5}=36$, $a_{1,M2}=0.0451$, $a_{1,VC}=2.66$, $a_{2,M2}=107$, and $a_{2,VC}=1.81$. At point 9, $b_{1}=1.9\times 10^{5}$, $b_{2}=1.13$, $b_{3}=22.5$, $b_{4}=356$, $b_{5}=36$, $a_{1,M2}=4.51$, $a_{1,VC}=26.6$, $a_{2,M2}=107$, and $a_{2,VC}=18.1$. On the *x*-axes, *n* is the integer in the wavenumber ($k=n\pi$). **b** is a magnified version of **a**

We ran our simulations at one of the ten Turing points given in Table 5. Although the last Turing point has the sharpest dispersion relation (Fig. 6d), it also has more than 40 unstable Fourier modes. It means a noisy and irregular pattern is likely to emerge at this point. The dispersion relations at the fifth and ninth Turing points from Table 5 are plotted in Fig. 9. They have fewer than ten unstable Fourier modes each. We simulated at the ninth point because the difference between the two diffusion rates is smaller; the ninth point is physically more likely. Therefore, the selected Turing point is defined as follows: $b_{1}=1.9\times 10^{5}$, $b_{2}=1.13$, $b_{3}=22.5$, $b_{4}=356$, $b_{5}=36$, $a_{1,M2}=4.51$, $a_{1,VC}=26.6$, $a_{2,M2}=107$, and $a_{2,VC}=18.1$.

We used COMSOL Multiphysics version 5.2, the finite element method software package, for our simulations. The finite element method involves discretising nonlinear differential equations and solving the resulting nonlinear algebraic equations numerically. We ran the simulations on a desktop computer with an Intel(R) Core(TM) i5-3570 CPU at 3.40 GHz and 16 GB of RAM. We adopted a fully coupled approach to solve Eqs. (13)–(18) simultaneously. We used the ‘constant (Newton)’ solver with a constant damping factor of 0.9. We specified that the algorithm had to terminate when the estimated relative error was less than 0.01, but we also set the maximum number of iterations in both space and time at 8. We allowed COMSOL to use the ‘PARDISO’ solver with a pivoting perturbation of $1\times 10^{-8}$ for linear problems; this solver tackles linear equations directly rather than iteratively.

In each simulation, we used the HSS concentrations plus noises as the initial concentrations. We used a random function to model these noises. It varies with the spatial coordinates, the number of which agrees with the number of dimensions; it is centred at 0 with an amplitude of 0.001. In a mesh, the function is evaluated at each node according to a uniform distribution. It means that a fine mesh mimics the randomness of noises more accurately than a coarse one. This feature prompted us to choose the ‘extremely fine’ mesh setting. It is the finest mesh setting available in COMSOL Multiphysics version 5.2: a nondimensionalised length of 1 is divided into 101 grid points and 100 even intervals.

In each simulation, we solved the model for ${\tilde{t}}$ ranging from 0 to 100. We used the BDF (backward differentiation formula) method to determine the time steps adaptively. In general, at each time point, this algorithm estimates the time derivatives of the next time step based on the solutions from the previous one or two time steps, and it determines the step size based on the derivatives’ stability. If the derivatives change drastically over the preceding time steps, the algorithm will recommend a small step. In our specific case of using the BDF method, we divided the ${\tilde{t}}$ range into 10,000 equal intervals and chose the ‘strict’ setting. The ‘strict’ setting means that a time step must end before or at the end of the interval it starts in. In our case, by using this setting, we effectively set a maximum time step of 0.01.

### One Dimension

**Fig. 10:**
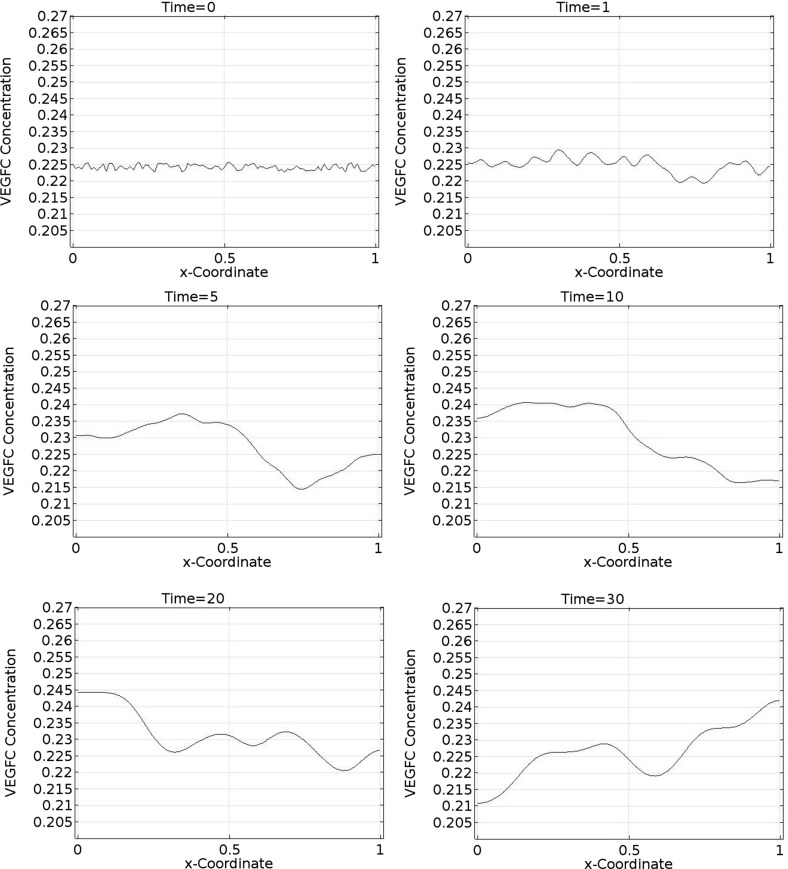
Spatiotemporal dynamics of VEGFC in one dimension (part 1). In this simulation, $b_{1}=1.9\times 10^{5}$, $b_{2}=1.13$, $b_{3}=22.5$, $b_{4}=356$, $b_{5}=36$, $a_{1,M2}=4.51$, $a_{1,VC}=26.6$, $a_{2,M2}=107$, and $a_{2,VC}=18.1$. VEGFC stands for vascular endothelial growth factor C

**Fig. 11:**
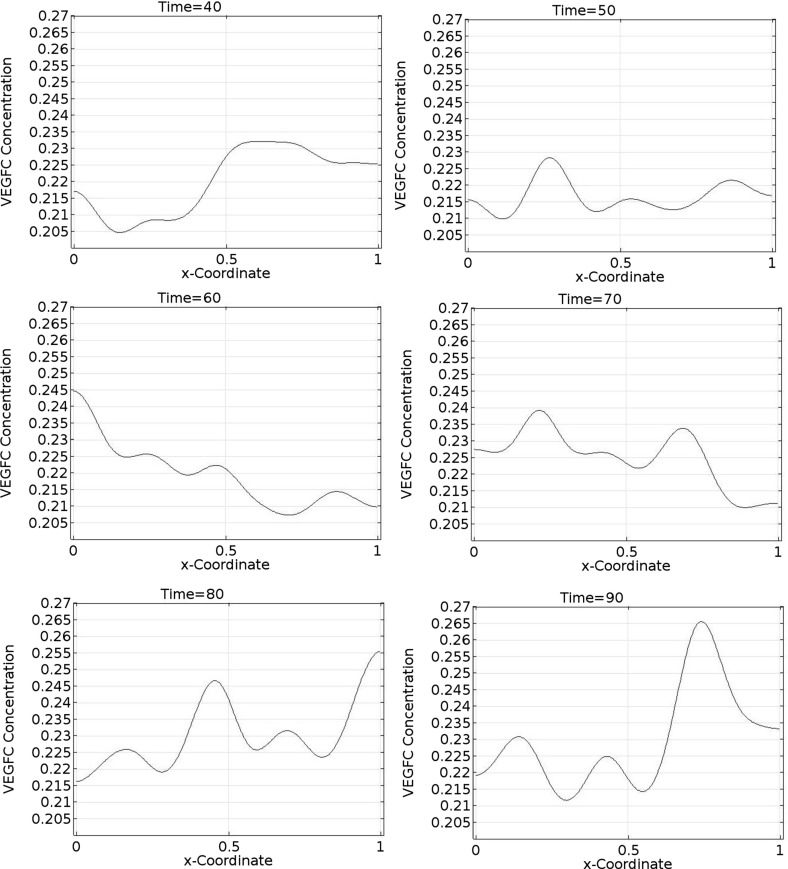
Spatiotemporal dynamics of VEGFC in one dimension (part 2). In this simulation, $b_{1}=1.9\times 10^{5}$, $b_{2}=1.13$, $b_{3}=22.5$, $b_{4}=356$, $b_{5}=36$, $a_{1,M2}=4.51$, $a_{1,VC}=26.6$, $a_{2,M2}=107$, and $a_{2,VC}=18.1$. VEGFC stands for vascular endothelial growth factor C

**Fig. 12:**
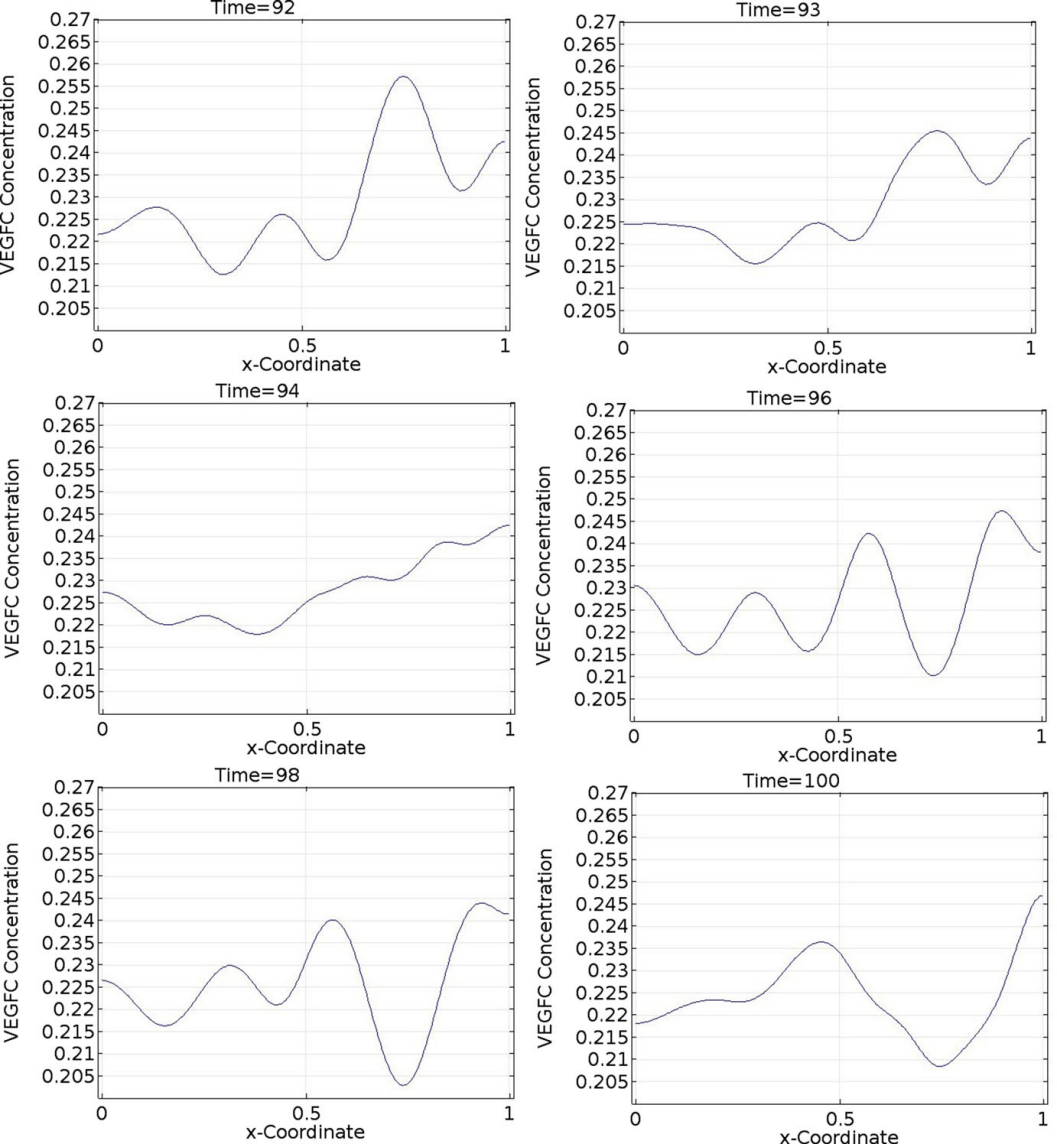
Spatiotemporal dynamics of VEGFC in one dimension (part 3). In this simulation, $b_{1}=1.9\times 10^{5}$, $b_{2}=1.13$, $b_{3}=22.5$, $b_{4}=356$, $b_{5}=36$, $a_{1,M2}=4.51$, $a_{1,VC}=26.6$, $a_{2,M2}=107$, and $a_{2,VC}=18.1$. VEGFC stands for vascular endothelial growth factor C

**Fig. 13:**
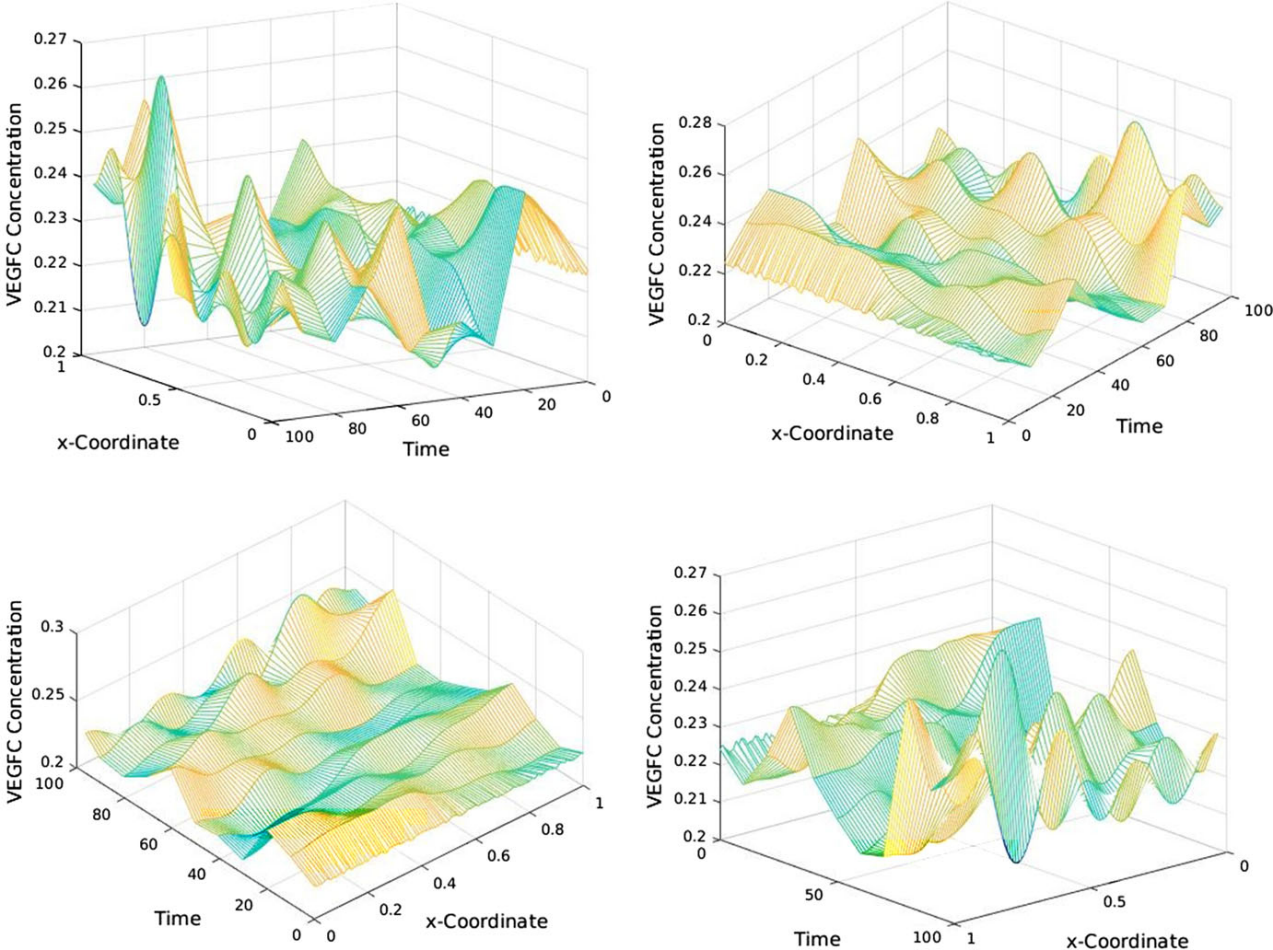
Spatiotemporal dynamics of VEGFC in one dimension (parts 1, 2, and 3). The four subfigures show the same results from different angles. In this simulation, $b_{1}=1.9\times 10^{5}$, $b_{2}=1.13$, $b_{3}=22.5$, $b_{4}=356$, $b_{5}=36$, $a_{1,M2}=4.51$, $a_{1,VC}=26.6$, $a_{2,M2}=107$, and $a_{2,VC}=18.1$. VEGFC stands for vascular endothelial growth factor C

Figures 10, 11, and 12 illustrate the spatiotemporal dynamics of VEGFC concentration in one spatial dimension. Figure 13 shows the same dynamics in three-dimensional plots.

Most noise components decay quickly. In Fig. 10, the third profile (${\tilde{t}}=5$) is considerably smoother than the first two profiles (${\tilde{t}}=0$ and ${\tilde{t}}=1$); the fluctuations present in the first profile are largely absent in the third.

On the other hand, the unstable components grow to broaden the concentration range. In Fig. 10, the concentration range in the final profile (${\tilde{t}}=30$) is an order of magnitude wider than that in the first profile (${\tilde{t}}=0$). The range in the final profile (${\tilde{t}}=90$) in Fig. 11 is even wider: twice the range in the case where ${\tilde{t}}=30$.

Eventually, three peaks emerge and vanish in an oscillatory manner, each time forming a more regular and clearer pattern. They first appear, albeit vaguely, in the second profile (${\tilde{t}}=50$) in Fig. 11. They vanish in the next two profiles (${\tilde{t}}=60$ and ${\tilde{t}}=70$) before reappearing, more prominently this time, in the last two profiles (${\tilde{t}}=80$ and ${\tilde{t}}=90$). This trend continues in Fig. 12. We note that in the fourth profile (${\tilde{t}}=96$) there, the peaks become closer in size than they are in the first profile (${\tilde{t}}=92$): a more regular pattern.

According to our prediction in the last section, the fastest growing Fourier mode at this Turing point has a wavelength of 0.333 (Table 5). The presence of three peaks is consistent because the domain size is 1. We tested our prediction more rigourously using the discrete Fourier transform in MATLAB R2012a. The transformed profile for the case where ${\tilde{t}}=96$ confirms our prediction.

To explain the oscillatory behaviour and growing concentration range, we must consider the eigenvalues of *B* more carefully. At the chosen Turing point and dominant wavenumber ($k=6\pi$), they are $-4.2756\times 10^{4}$, $-1.1887$, $1.0570\times 10^{-2}-0.4510j$, and $1.0570\times 10^{-2}+0.4510j$. The two eigenvalues with positive real parts are a pair of complex conjugates, hence the oscillations and growing concentration range.

When the three peaks are present, they have different widths and heights. There are two reasons. First, there is more than one unstable Fourier mode; they are interruptions to the dominant one. Second, we only carried out a linear stability analysis; we cannot predict the peak amplitudes without considering the nonlinear dynamics.

In summary, Turing’s mechanism does work at the Turing point considered in the simulation. However, if we take the purist’s perspective and insist that Turing patterns are stationary, nonlinear, and spatially periodic, the simulated VEGFC patterns are not Turing patterns. The aforementioned control mechanism must not only create the physiological conditions at the Turing points as defined in Sect. 3.5, but also select the conditions compatible with stationary patterns.

### Two Dimensions

Clearly, the zebrafish is not a line. The geometry we used in our previous study has an aspect ratio around 10 (Wertheim and Roose 2017). We repeated the simulation presented in the last subsection in a rectangle with an aspect ratio of 10. To extend our model to two dimensions, we replaced each spatial derivative with respect to $\tilde{x}$ with the gradient operator ($\nabla$). The results are not shown because they are equivalent to those in the last subsection: as time passes, three VEGFC-rich regions emerge and vanish in an oscillatory manner.

**Fig. 14:**
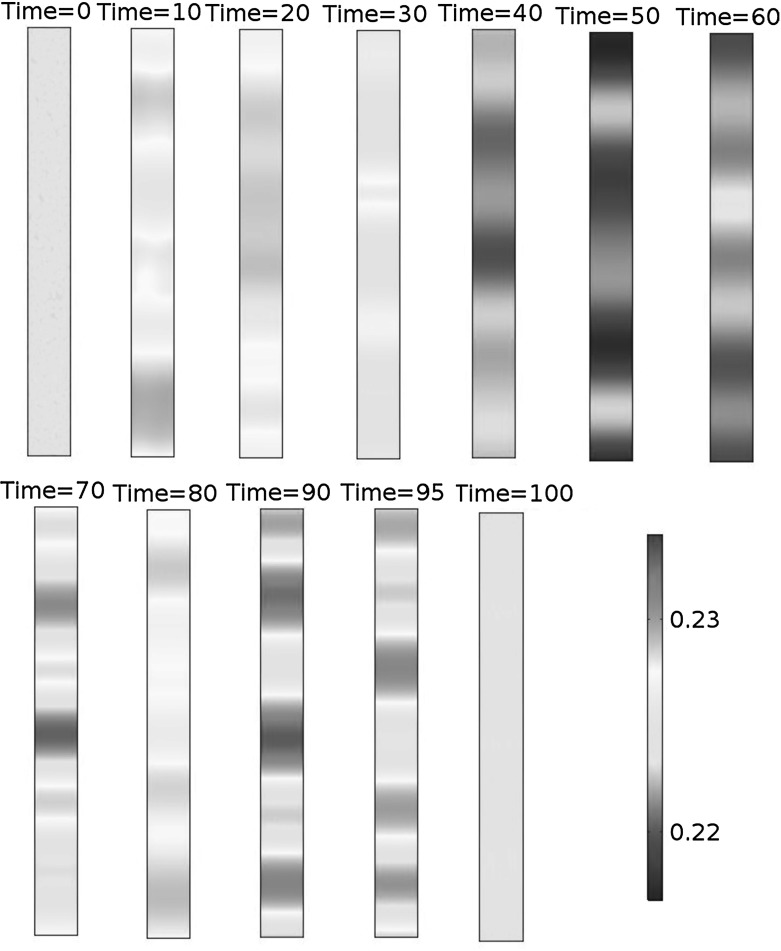
Spatiotemporal dynamics of VEGFC in two dimensions and with periodic boundary conditions. In this simulation, $b_{1}=1.9\times 10^{5}$, $b_{2}=1.13$, $b_{3}=22.5$, $b_{4}=356$, $b_{5}=36$, $a_{1,M2}=4.51$, $a_{1,VC}=26.6$, $a_{2,M2}=107$, and $a_{2,VC}=18.1$. Vertically, the spatial coordinate $\tilde{y}$ goes from 0 to 1; horizontally, the spatial coordinate $\tilde{x}$ goes from 0 to 0.1. VEGFC stands for vascular endothelial growth factor C

We ended this study by experimenting with the boundary conditions. We replaced the no-flux boundary conditions with periodic boundary conditions. The geometry has two pairs of opposing boundaries, so we made each pair periodic. The amended model is different in two ways. First, each concentration on a boundary mirrors its counterpart on the opposite boundary. Second, an influx of VEGFC or MMP2 through a boundary is balanced by an outflux through the opposite boundary. With the new boundary conditions in place, the solution to Eq. (30) is proportional to $\sum \nolimits _{n=0}^{\infty }e^{\sigma _{n}{\tilde{t}}}\Big [\cos (k\tilde{x})+\sin (k\tilde{x})\Big ]$. However, the dispersion relations remain the same, meaning the results presented in the last two sections are still valid. The simulation results obtained with periodic boundary conditions are illustrated in Fig. 14. Figure 14 has two notable features. First, the distribution of VEGFC is very regular in Fig. 14. Second, the concentration range in Fig. 14 is narrower than those in Figs. 10, 11, and 12. The increased regularity is consistent with the use of periodic boundary conditions, while diffusion in two dimensions leads to flatter concentration gradients than it does in one dimension. These two features aside, the comments on the other two simulations apply to Fig. 14 too.

Clearly, the outer boundaries of the trunk are physical barriers and the trunk is not periodic in the two modelled directions. The system with periodic boundary conditions represents a portion of the trunk, far from and not influenced by the boundaries. Therefore, this patterning mechanism is versatile.

## Discussion

At the start of this study, we wondered what guides the differentiation of PLs into the mature LECs that form the TD, DLLV, and PLV. We wondered whether VEGFC, MMP2, and collagen I can interact to generate Turing patterns of VEGFC. We have shown that the answer is ‘yes’ and analysed the patterning mechanism (Sect. 4). On the other hand, only 94 of the 1,953,125 Turing point candidates are Turing points. It is obvious that the zebrafish embryo needs a separate control mechanism to create the supporting physiological conditions. We found out the factors that may favour Turing’s mechanism, insights into what the control mechanism needs to achieve (Sect. 4). Finally, we buttressed our predictions with computer simulations (Sect. 5). To end this paper, we will link the proposed patterning mechanism to lymphangiogenesis.

The patterning mechanism requires a HSS. The embryo is closer to a HSS after the migration of MMP2-producing PLs is over. On this basis, we argue that the PLs from the horizontal myoseptum migrate to where the vessels lie before differentiating into mature LECs. In other words, the TD, DLLV, and PLV form and then mature. It follows that the differentiation/maturation step, regulated by the proposed patterning mechanism, occurs at the tail end of the time frame, closer to 168 HPF than 48 HPF.

However, it is premature to relate this patterning mechanism to the maturation of the three lymphatic vessels. First, the peaks of VEGFC concentration may not coincide with the vessels. Second, if VEGFC demonstrates the oscillatory behaviour present in the simulations, it will be an inconsistent signal. Third, in the simulation results, the concentration range of VEGFC is less than threefold, the prerequisite of a morphogen gradient according to a rule of thumb (Gurdon and Bourillot 2001). Fourth, the nondimensionalised time range in our simulations corresponds to almost 12 days. Considering the zebrafish reaches sexual maturity at roughly 3 months post-fertilisation (Nasiadka and Clark 2012), the simulated dynamics may be too slow.

We need more information to forge the links. First, we must explain what makes the vessels lie at the peaks of a VEGFC pattern. It is possible that the constituent cells are drawn there by chemotaxis. Although we express scepticism about this scenario in our earlier paper (Wertheim and Roose 2017), the study reported in that paper was about an earlier stage of lymphangiogenesis. Besides, that argument rests on the distribution of VEGFC in the trunk, not its intrinsic ability to chemoattract LECs or their progenitors. Second, we must explore the parametric space more selectively. We should only pick the parametric combinations whose dominant Fourier modes have real and sufficiently large eigenvalues. At these Turing points, stationary VEGFC patterns reach a sufficient size within a reasonable time frame. Biologically, it means the aforementioned control mechanism must be selective enough to pick the physiological conditions representing these Turing points over those representing the other Turing points.

Focussing on the study itself, we can make certain improvements. The most obvious criticism pertains to the aforementioned control mechanism. While we have shown that VEGFC *can* form Turing patterns, it is of no use to the zebrafish embryo unless this ability is exercised. The patterning mechanism needs the control mechanism. Therefore, we must explain how the embryo finds the ‘right’ parameters by carrying out a more extensive and sophisticated search of the parametric space. For example, an adaptive Monte Carlo algorithm can mimic how the embryo may find the right parameters by trial and error. Then, using a larger sample of Turing points, we can look for correlations between the 11 parameters to infer a mechanism. A fundamental assumption is the existence of a HSS. To provide evidence for it, we must track the biochemical profile in the zebrafish embryo over time. The MMP2 and VEGFC production terms are essential to Turing’s mechanism. In order to address the uncertainties in these terms, we must perform experimental studies.

However, even before we find the missing links or improve the study, our results are novel and useful. Although Turing’s mechanism was first proposed decades ago, our biochemical system is an atypical instance due to the production terms, the interactions of VEGFC with an immobile substrate, and the dependence of diffusion rates on the abundance of immobile substrate. To the best of our knowledge, no one has performed a linear stability analysis on this type of biochemical system before. Therefore, we have proposed a new patterning mechanism. It has two indispensable components: reversible binding between VEGFC and collagen I (positive feedback for VEGFC) and VEGFC-enhanced production of MMP2, which degrades collagen I (negative feedback for VEGFC). We also know that volume exclusion by collagen I hinders rather than promotes pattern formation. We have shown that the mechanism works for both periodic and no-flux boundary conditions. Therefore, it can be applied to other natural and synthetic biological systems.
